# Baf53a is involved in survival of mouse ES cells, which can be compensated by Baf53b

**DOI:** 10.1038/s41598-017-14362-4

**Published:** 2017-10-25

**Authors:** Bo Zhu, Atsushi Ueda, Xiaohong Song, Shin-ichi Horike, Takashi Yokota, Tadayuki Akagi

**Affiliations:** 1 Department of Stem Cell Biology, Faculty of Medicine, Institute of Medical, Pharmaceutical and Health Sciences, Kanazawa University., 13-1 Takara-machi, Kanazawa, Ishikawa, 920-8640 Japan; 2 Advanced Science Research Center, Kanazawa University. 13-1 Takara-machi, Kanazawa, Ishikawa, 920-8640 Japan

## Abstract

The human Baf (Brg1/Brm associated factor) complex, also known as the mammalian SWI/SNF chromatin-remodeling complex, is involved in a variety of cellular processes. The pluripotency and self-renewal abilities are major characteristics of embryonic stem (ES) cells and are regulated by the ES cell-specific BAF (esBAF) complex. Baf53a is one of the subunits of the esBAF complex. Here, we found that Baf53a was expressed in undifferentiated ES cells and that it interacted with Oct3/4. Analyses of tetracycline-inducible Baf53a conditional knockout ES cells revealed that the undifferentiated markers, including Nanog and Oct3/4, were expressed in Baf53a-deficient ES cells; however, growth of the cells was repressed, and expression of p53, p21, and cleaved Caspase 3 was increased. Cell death of Baf53a-deficient ES cells was rescued by overexpression of Baf53a, but not by the Baf53a M3 mutant (E388A/R389A/R390A). Interestingly, Baf53b, a homologue of Baf53a, rescued cell death of Baf53a-deficient ES cells. Baf53a-deficient ES cells overexpressing exogenous Baf53a or Baf53b remained in the undifferentiated state, proliferated, and repressed expression of p21. In summary, our findings suggest that Baf53a is involved in the survival of ES cells by regulating p53 and Caspase3, and that Baf53b is able to compensate for this functional aspect of Baf53a.

## Introduction

Mouse embryonic stem (ES) cells exhibit self-renewal capability and pluripotency. These abilities are maintained in the presence of leukemia inhibitory factor (LIF), which induces the intracellular JAK/STAT3 signaling pathway. Mouse ES cells are established from preimplantation embryos and maintain their full developmental potential in the presence of LIF; mouse ES cells are thus thought to exist in a naïve state. The ground state in naïve ES cells is achieved by the addition of inhibitors for the GSK3 and Erk signaling pathways in the culture medium^1–3^. Pluripotency of mouse ES cells is regulated by several transcription factors. Previously, we and others demonstrated that STAT3 activation is sufficient for the maintenance of undifferentiated status^4,5^, and Oct3/4 is one of the major regulators of pluripotency^6^. Based on these findings, we have identified several downstream targets of STAT3 and/or Oct3/4, or Oct3/4-interacting proteins. These include Zfp57, Eed, Dax1, Esrrb, Zfp296, ETV4/5 and so on^7–13^. Furthermore, an extended gene regulatory network tightly controls differentiation and proliferation of ES cells and participates in establishment of the pluripotency^14–16^. Recent computational analysis suggests that sets of defined transcription factors could generate naïve pluripotency of ES cells^17^.

In addition to transcription factors, chromatin regulators are also involved in the regulation of pluripotency in ES cells^18^. The Brg1/Brm-associated factors (Baf) complex, which is also known as the mammalian SWI/SNF ATP-dependent chromatin-remodeling complex, is known to affect the differentiation of embryonic and adult stem cells. A core factor of the complex, either Brg1 or Brm, is required for these processes^19–23^. The Baf complex consists of several subunits^24^ and compositions of the complexes are varied among each cell type. For example, Baf45a and Baf53a are subunits of a complex in neural stem/progenitor cells, which is known as the neural stem/progenitor BAF (npBAF) complex^25^. In post-mitotic neuronal cells, these two subunits are replaced with Baf45b/c and Baf53b, respectively, forming a neuron-specific BAF (nBAF) complex^25^. Embryonic stem cell-specific BAF (esBAF) complex consists of several subunits, including Brg1, Baf155, Baf60a, and Baf45d, and interacts with pluripotency-regulating transcription factors^26^. While the LIF/STAT3 signaling is the major regulator to maintain pluripotency in mouse ES cells, one of the polycomb group complexes, the polycomb repressive complex 2 (PRC2), enhances H3K27me3 to repress differentiation-associated gene expression. Brg1 of the esBAF complex is involved in the establishment of chromatin accessibility at STAT3 binding target sites and in the regulation of PRC2 function, thereby regulating the pluripotency in ES cells^27^. Importantly, the catalytic core component of Brg1 is required for the functional regulations of the esBAF complex. In addition, each subunit of the complex have critical functions that mediate physiological responses in ES cells, for example, Baf155, Baf250a, and Baf250b are known to regulate proliferation and differentiation in mouse ES cells^28–31^.

Baf53a (also known as Actl6a or Arp4) is one of the subunits that make up the npBAF and esBAF complexes and is expressed in several stem/progenitor cells, including neural progenitor cells, hematopoietic stem cells, epidermal progenitor cells, and ES cells. Forced expression of Baf53a together with Baf45a in neuronal progenitor cells prevented differentiation. When Baf53a was knocked down in neural progenitor cells, proliferation was impaired, indicating that Baf53a was required for proliferation of neural stem/progenitor cells^25^. Conditional knockout (cKO) of Baf53a in hematopoietic stem cells (HSCs) resulted in mice with bone marrow failure, aplastic anemia, and rapid death. Cell counts revealed a decrease in mature hematopoietic cells (*e.g*., macrophages, granulocytes, erythrocytes, B cells and T cells) and HSCs/progenitor cell fractions (*e.g*., common myeloid progenitors, megakaryo-erythrocyte progenitors, granulocyte-monocyte progenitors, and c-kit^+^ Lin^−^ Sca-1^+^ cells) in Baf53a cKO bone marrow, indicating an involvement of Baf53a in the proliferation and survival of hematopoietic cells^32^. Conditional KO of the Baf53a gene in epidermis cells resulted in cell cycle exit, terminal differentiation, and hypoplasia, whereas ectopic expression of Baf53a repressed Klf4 expression and enhanced epidermal progenitor state, suggesting that Baf53a prevented differentiation of epidermal progenitor cells^33^. Recently, Lu *et al*., demonstrated that knockdown of Baf53a expression by RNAi in ES cells induced reduction of pluripotent marker genes (Nanog and Oct3/4) and subsequent differentiation into primitive endoderm^34^. In summary, these observations suggest that Baf53a regulates characteristics typical of stem/progenitor cells, such as proliferation, differentiation, survival, and cell-fate determination. In the present study, we generated tetracycline (Tet)-inducible Baf53a cKO ES cells and found that Baf53a is involved in survival of ES cells.

## Results

### Expression of Baf53a in ES cells and interaction between Baf53a and Oct3/4

**Figure 1:**
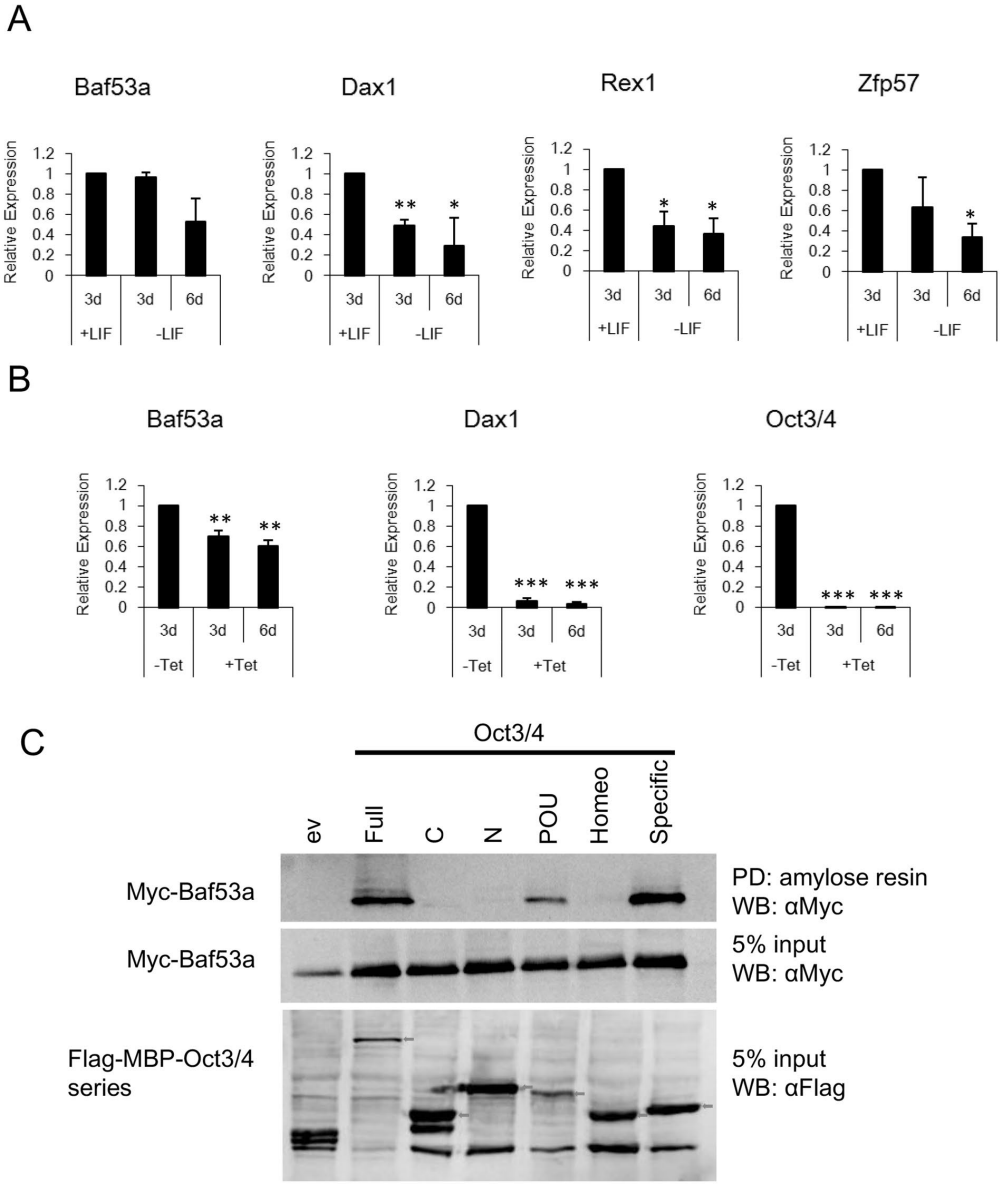
Baf53a is expressed in ES cells, and Baf53a interacts with the POU-specific domain of Oct3/4. (**A**) Expression of Baf53a mRNA in E14 ES cells cultured with or without LIF for 3–6 days. Expression of Baf53a and markers of undifferentiated cells (Dax1, Rex1, and Zfp57) mRNA was examined by quantitative RT-PCR. (**B**) Expression of Baf53a mRNA after downregulation of Oct3/4. ZHBTc4 ES cells were cultured with or without 1 μg/mL tetracycline (Tet) for 3–6 days. Expression of Baf53a mRNA was examined by quantitative RT-PCR. Dax1 was used for a positive control as an Oct3/4 target gene. These data were normalized to GAPDH, and the results are expressed as the average of three independent experiments ± the standard deviation. *p < 0.05, **p < 0.01, ***p < 0.001 (t-test). (**C**) Interaction between Baf53a and Oct3/4. The Myc-Baf53a expression vector was co-transfected with Flag-MBP-Oct3/4 or its truncated mutants, including C-terminal region, N-terminal region, POU domain, POU-homeo domain, and POU-specific domain, into HEK293 cells. Flag-MBP-fused proteins were pulled down using amylose resin, and the precipitates were examined by Western blot with anti-Myc antibody. Expression of each protein was confirmed with anti-Myc and anti-Flag antibodies. Arrows indicate Flag-MBP-Oct3/4 and its truncated mutants. The result shown is representative of three independent experiments. Note: ev = control Flag-MBP empty vector; Full = full length of Oct3/4 (1–352 aa); C = C-terminal region (283–352 aa); N = N-terminal region (1–130 aa); POU = POU domain (131–282 aa); Homeo = POU-homeo domain (217–282 aa); Specific = POU-specific domain (131–213 aa).

We first examined the expression of Baf53a mRNA in wild-type E14 ES cells and in ZHBTc4 (Tet-inducible, Oct3/4 cKO) ES cells. Quantitative RT-PCR analysis revealed that expression of Dax1, Rex1, and Zfp57 (undifferentiated state marker genes) in E14 ES cells was decreased after removal of LIF from the culture medium. In contrast, expression level of Baf53a mRNA in cell samples cultured in -LIF for 3 days was comparable to that of + LIF condition; Baf53a was reduced at 6 days after removal of LIF (Fig. 1A). Expression of Oct3/4, as well as its downstream target gene, Dax1, was completely repressed by Tet stimulation in ZHBTc4 ES cells (Fig. 1B). Expression of Baf53a was decreased by Tet stimulation, but its reduction was milder than the changes observed for Dax1 expression. Taken together, these results show that Baf53a is expressed in undifferentiated ES cells, and its expression is retained under our differentiation condition.

Baf53a is one of the subunits of the esBAF complex, and Brg1, a core factor of the complex, is known to associate with Oct3/4 ^26^. Baf53a is one of the candidate molecules predicted to also interact with Oct3/4 ^35^, thus, the esBAF complex is thought to be involved in the regulation of the pluripotent state of ES cells. We therefore examined whether Baf53a interacts with Oct3/4. Maltose binding protein (MBP) pulldown assay showed that full-length form of Oct3/4, as well as the POU and POU-specific domains of Oct3/4 interacted with Baf53a (Fig. 1C). Thus, it appears that Oct3/4 can associate with the esBAF complex via both Baf53a and Brg1.

### Baf53a knockdown in ES cells results in expression of markers of undifferentiated cells and reduction of cell numbers

As described above, we discovered expression of Baf53a in ES cells even in differentiating conditions and interaction with Oct3/4 via POU domain, indicating a crucial function(s) of Baf53a in ES cells. To explore Baf53a function in ES cells, first we performed knockdown (KD) analyses. Baf53a KD experiments using two independent Baf53a siRNAs (siBaf53a #1 and #2) revealed that morphological signatures and alkaline phosphatase (AP) activities were comparable between control and Baf53a KD ES cells (Supplemental Fig. 1A). In addition, expression analysis showed that endogenous expression of Baf53a mRNA and protein was effectively reduced by the treatment with siRNAs; however, expression of the pluripotent state markers, Oct3/4, Dax1, Esrrb, and Nanog, was sustained in Baf53a KD ES cells (Supplemental Fig. 1B,C). These results indicate that Baf53a does not regulate the undifferentiated state in ES cells.

During the knockdown experiments, we noticed that proliferation was repressed in Baf53a KD ES cells. Indeed, the WST-1 assay revealed that growth of Baf53a KD ES cells were obviously reduced compared with control ES cells (Supplemental Fig. 1D).

### Baf53a deficiency induces p53 and p21 expression and activation of Caspase 3

**Figure 2:**
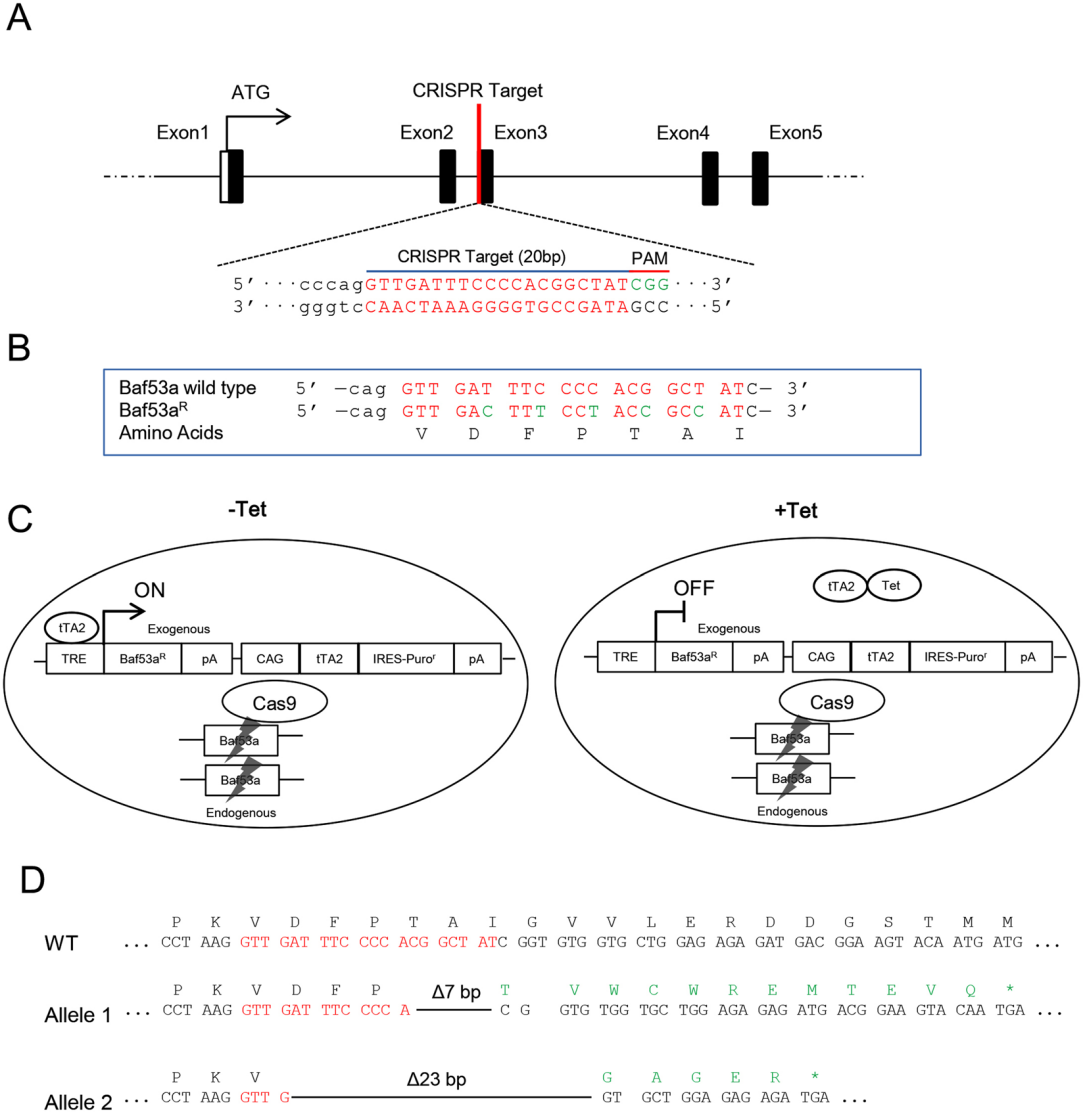
Strategy for generation of tetracycline (Tet)-inducible Baf53a conditional knockout (cKO) ES cells. (**A**) The targeting site of Baf53a. The targeting site (5′-GTT GAT TTC CCC ACG GCT AT-3′) of the Baf53a gene was shown. The target sequences are located on Exon 3 of the gene. (**B**) Alignment of the target sites of wild-type Baf53a and CRISPR/Cas9 resistant Baf53a (Baf53a^R^). The targeting site of the Baf53a cDNA was changed to 5′-GTT GAc TTt CCt ACc GCc AT-3,’ which encodes an identical amino acid sequence. (**C**) Schematic view of Tet-inducible Baf53a cKO ES cells. Expression of exogenous Baf53a^R^ was induced in the absence of Tet and repressed in the presence of Tet. Both alleles of the Baf53a gene were mutated by the CRISPR/Cas9 system. (**D**) Sequences of deleted alleles. Genomic DNA of the deleted regions was sequenced. One allele contained a 7 bp deletion and the other contained a 23 bp deletion.

**Figure 3:**
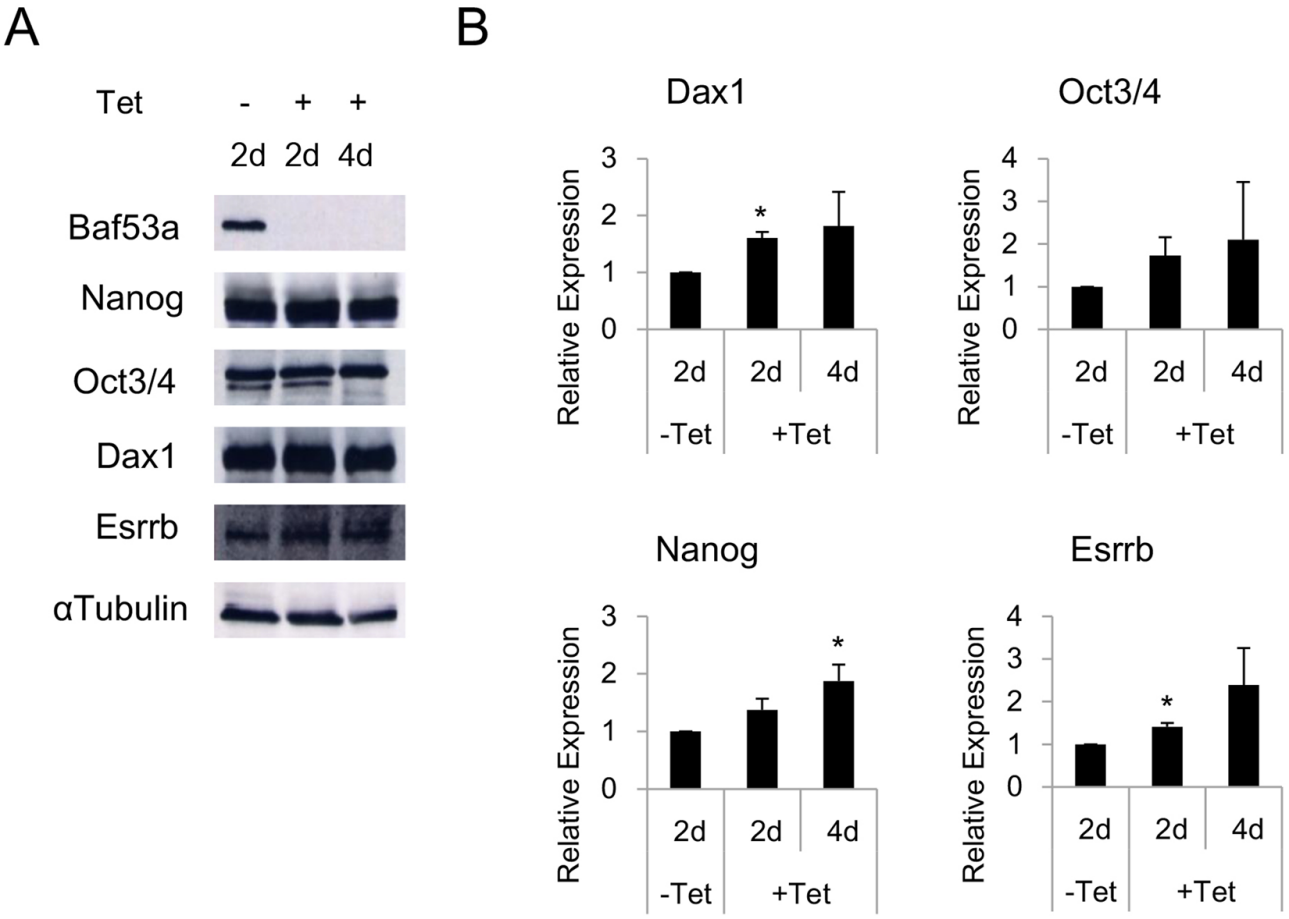
Expression of undifferentiated state marker genes in Baf53a-deficient ES cells. (**A**) Protein expression of markers characteristic of the undifferentiated state was analyzed by Western blot in tetracycline (Tet)-inducible Baf53a conditional knockout (cKO) ES cells. Baf53a cKO ES cells were cultured with or without 1 μg/mL Tet for 2–4 days. αTubulin was used as a loading control. The data is representative of three independent experiments. (**B**) Expression of Dax1, Oct3/4, Esrrb, and Nanog mRNA was examined by quantitative RT-PCR. The data was normalized to GAPDH expression, and the results are the means ± standard deviations of three independent experiments. *p < 0.05 (t-test).

To better characterize the functions of Baf53a in ES cells, we generated Tet-inducible Baf53a cKO ES cells using the CRISPR/Cas9 system. In this new cell line, the Baf53a gene was mutated by the CRISPR/Cas9 system, and the expression of exogenous CRISPR/Cas9-resistant Baf53a mutant was regulated by Tet (for details, see Materials and Method section) (Fig. 2). As shown in Fig. 3A, expression of Baf53a protein was successfully repressed after stimulation with Tet. The undifferentiated state marker genes, Nanog, Oct3/4, Dax1, and Esrrb, were expressed even after 4 days of inducing Baf53a knockout (Fig. 3A,B), which was in agreement with the knockdown experiments.

**Figure 4:**
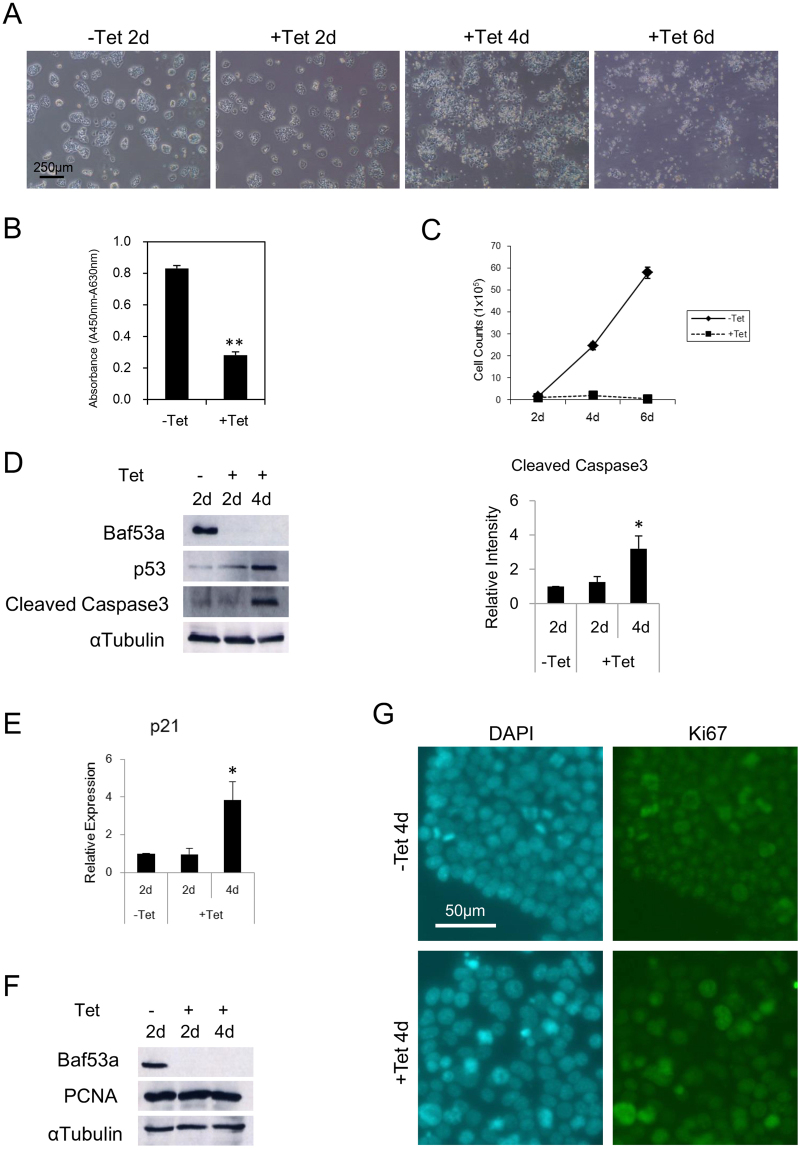
Viability of Baf53a-deficient ES cells is repressed. (**A**) Baf53a cKO ES cells were cultured for 2–6 days in 1 μg/mL Tet and their morphologies were examined on the days indicated. Scale bar is 250 μm. (**B**) Baf53a cKO ES cells were seeded at 1000 cells per well in a 96-well dish, cultured with or without 1 μg/mL Tet, and subjected to the WST-1 assay after 4 days. The data represents three independent experiments and the means ± standard deviations are shown. **p < 0.01 (t-test). (**C**) Growth curves of Baf53a cKO ES cells. Baf53a cKO ES cells were cultured in the presence or absence of 1 μg/mL Tet and the number of cells was counted on the days indicated. The result is the means ± standard deviations of three independent experiments. (**D**) Baf53a cKO ES cells were cultured with or without 1 μg/mL Tet for 2–4 days, and protein expression of Baf53a, p53, and cleaved Caspase 3 was examined by Western blot analysis. αTubulin was used as a loading control. The data is representative of three independent experiments (left panel). Signal intensity of bands of cleaved Caspase 3 was measured by a densitometry and the result is the means ± standard deviations of three independent experiments (right panel). *p < 0.05 (t-test). (**E**) Expression of p21 mRNA was examined by quantitative RT-PCR. The data was normalized to GAPDH expression, and the result is the means ± standard deviations of three independent experiments. *p < 0.05 (t-test). (**F**) Baf53a cKO ES cells were cultured with or without 1 μg/mL Tet for 2–4 days, and protein expression of Baf53a and PCNA was examined by Western blot analysis. αTubulin was used as a loading control. The data is representative of two independent experiments. (**G**) Baf53a cKO ES cells were cultured with or without 1 μg/mL Tet for 4 days, and protein expression of Ki67 was examined by immunofluorescent stain. Nuclei were stained with DAPI. Scale bar is 50 μm. The data is representative of two independent experiments.

We found that proliferation was repressed when Baf53a expression was knocked down in ES cells; we therefore examined the rate of proliferation in Baf53a cKO ES cells. Morphological signatures showed that the colony number began to decrease after 4 days of Tet-treatment, and the majority of the cells underwent cell death by 6 days (Fig. 4A). WST-1 assay showed that proliferation of Tet-treated Baf53a cKO ES cells at 4 days was repressed (Fig. 4B), and cell counts revealed that cell growth of Baf53a cKO ES cells were significantly repressed in the presence of Tet (Fig. 4C). These observations indicate that Baf53a deficiency results in reduction of cell viability in ES cells.

Furthermore, we examined the molecular events underlying the reduction of viability in Baf53a-deficient ES cells. Western blot analysis showed that expression of p53 protein was detected in Tet-treated Baf53a cKO ES cells at 4 days, and the expression level of p21 mRNA, which is the downstream target of p53, was induced at day 4 (Fig. 4D,E). We also found induction of cleaved Caspase 3 in the Baf53a-deficient ES cells, and expression level of cleaved Caspase 3 in the Baf53a-deficient ES cells is 3-fold higher than control cells (Fig. 4D). In addition, we examined expressions of Ki67 and PCNA (proliferation markers) by immunofluorescent stain and Western blot analysis, respectively. As shown in Fig. 4F, Baf53a-deficient ES cells expressed PCNA. Immunofluorescent stain revealed that Baf53a-deficient ES cells are Ki67-positive (Fig. 4G). These results show that repression of Baf53a expression leads to the accumulation of p53 protein and activation of Caspase 3, which in turn causes growth repression and apoptosis in Baf53a-deficient ES cells, respectively, indicating that Baf53a is required for sustaining survival of ES cells.

### Wild-type Baf53a, but not Baf53a M3, rescues the viability of Baf53a-deficient ES cells

**Figure 5:**
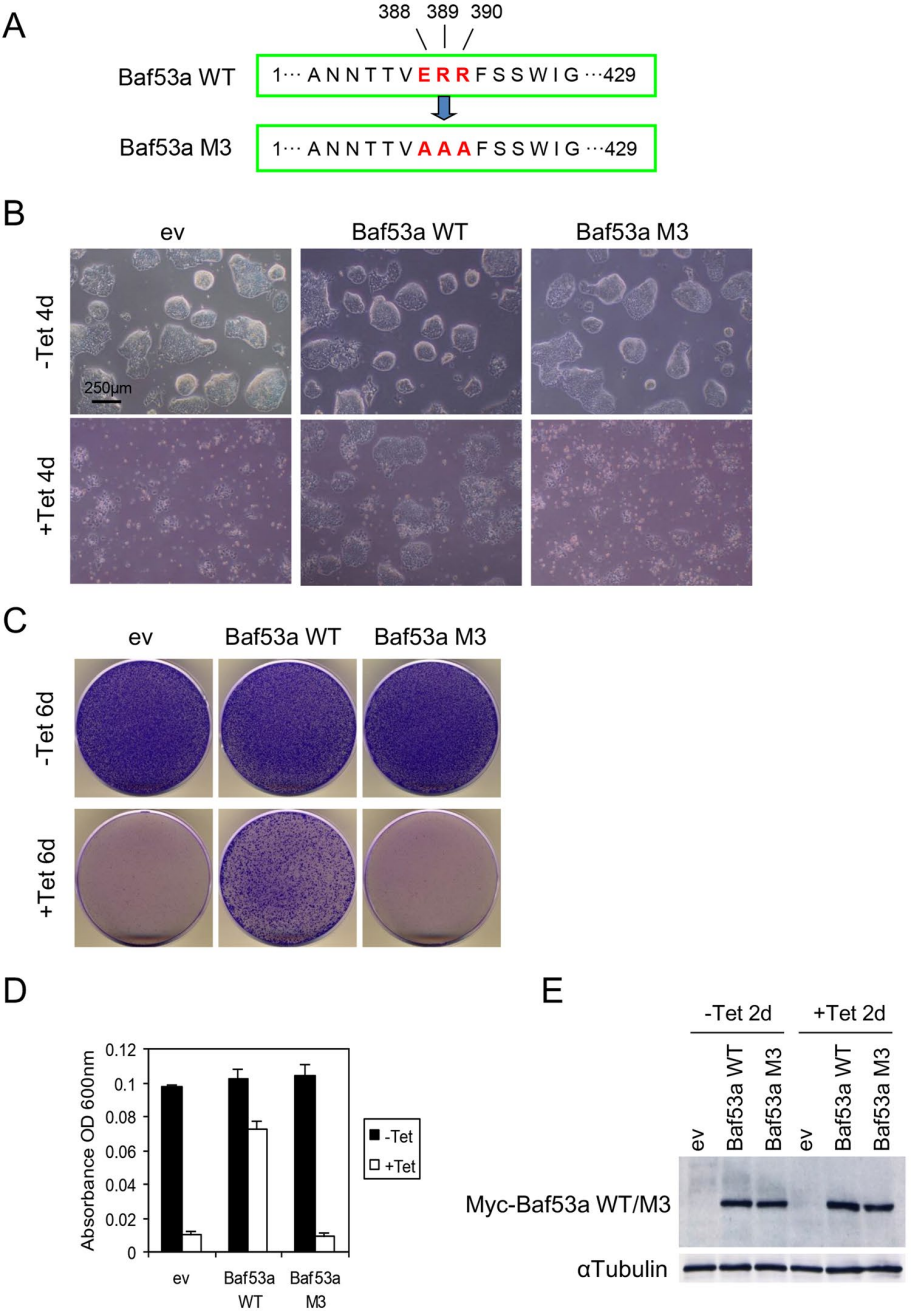
Transfection of wild-type Baf53a (WT), but not mutant Baf53a (M3), rescues the viability of Baf53a-deficient ES cells. (**A**) Schematic view of Baf53a M3 mutant. Three amino acid residues (glutamic acid (E), arginine (R), and arginine (R)) of wild-type Baf53a were changed into three alanine (A) residues. (**B**) Morphological signatures of Baf53a-expressing Baf53a cKO ES cells. Baf53a cKO ES cells were transfected with expression vectors encoding empty (ev), Baf53a WT, or Baf53a M3. Each transfected ES cells were divided into two dishes at 48 h after transfection and cultured with or without 1 μg/mL Tet for 4 days and their cell morphologies were observed. Scale bar is 250 μm. (**C**,**D**) Colony formation assay. (**C**)Baf53a WT- or Baf53a M3-expressing Baf53a cKO ES cells and control cells described in panel B were cultured for 6 days with or without 1 μg/mL Tet; and these samples were stained by crystal violet. (**D**) These colonies were dissolved in 1.0% SDS solution and absorbance was measured at 600 nm to quantify the number of surviving colonies. The representative results shown are expressed as the means ± standard deviation of three independent experiments. (**E**) Expression of exogenous Myc-tagged Baf53a protein. Expression of either Myc-Baf53a WT or Myc-Baf53a M3 was confirmed by Western blot at 2 days after Tet treatment. αTubulin was used as a loading control. The result is representative of three independent experiments.

The results above led us to examine whether overexpression of Baf53a in Baf53a-deficient ES cells could rescue the reduced proliferation phenotype. To test this, we transfected either a control empty vector (ev) or Myc-tagged wild-type Baf53a expression vector (Baf53a WT) into Baf53a cKO ES cells; 2 days after transfection, cells were divided into two dishes and cultured for an additional 4–6 days in the presence or absence of Tet. As shown in Fig. 5B, Baf53a cKO ES cells expressing ev failed to form ES cell-colonies in the presence of Tet; in contrast, Baf53a cKO ES cells expressing Baf53a WT formed colonies after 4 days of Tet-treatment. Cell survival was assessed using Crystal violet staining and quantified by measuring the absorbance of the cell lysates; these two assays indicated that colony forming cells were recovered in Baf53a cKO ES cells that overexpressed Baf53a WT in the presence of Tet (Fig. 5C,D). These data suggest that overexpression of Baf53a WT is sufficient to rescue the survival of Baf53a-deficient ES cells.

Baf53a is known to associate with β-actin, c-Myc, Brg1, and Tip60. Recently, the region responsible for mediating the interaction between Baf53a and other proteins was determined by constructing a series of Baf53a mutants (M1 to M8 mutations)^36^. From this mutational analysis, the M3 mutant (E388A/R389A/R390A) (Fig. 5A) was identified to be the most effective in disrupting protein interactions. Therefore, we examined whether Baf53a M3 mutant could rescue the phenotype of Baf53a-deficient ES cells. Interestingly, examination of morphological characteristics and the results from the colony forming assay revealed that Baf53a M3-expressing Baf53a cKO ES cells failed to form colonies after Tet treatment (Fig. 5B–D). Expression of exogenous Myc-tagged Baf53a WT and Baf53a M3 protein were comparable between these samples (Fig. 5E). These data show that Baf53a M3 had lost its original functions, and that the amino acid residues 388, 389, and 390 are all important for the full functioning of Baf53a. We also generated Baf53a WT- or Baf53a M3-overexpressing ES cells and examined proliferation of them by WST-1 assay. As shown in Supplemental Fig. 2A,B, overexpression of either Baf53a WT or M3 did not enhance proliferation of ES cells, indicating that Baf53a M3 does not have dominant negative activity.

### Artificial expression of Baf53b rescues cell death exhibited by Baf53a-deficient ES cells

**Figure 6:**
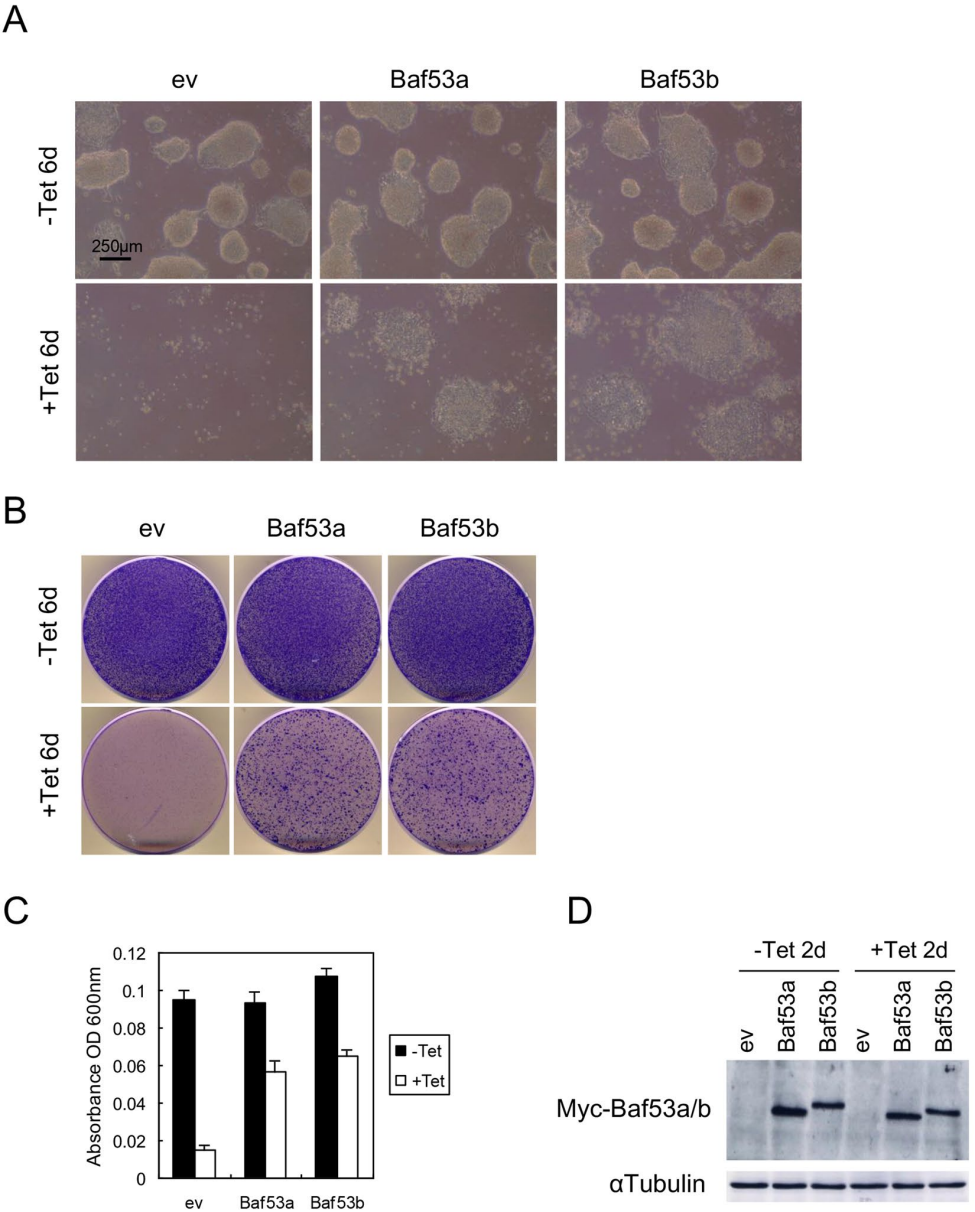
Expression of Baf53b rescues the survival of Baf53a-deficient ES cells. (**A**) Morphological signatures of Baf53b-expressing Baf53a cKO ES cells. Baf53a cKO ES cells were transfected with expression vectors for empty (ev), Baf53a, or Baf53b. Transfected ES cells were divided into two dishes at 48 h after transfection and cultured with or without 1 μg/mL Tet. Cell morphologies were observed after 6 days. Scale bar is 250 μm. (**B**,**C**) Colony formation assay. (**B**) Control and Baf53a- or Baf53b-expressing Baf53a cKO ES cells as described in panel A were cultured for 6 days with or without 1 μg/mL Tet; and these samples were stained with crystal violet. (**C**) The colonies were dissolved in 1.0% SDS solution and absorbance was measured at 600 nm to quantify the number of surviving colonies. The representative results shown are expressed as the means ± standard deviation of three independent experiments. (**D**) Expression of exogenous Myc-tagged Baf53a and Baf53b protein. Expression of either Myc-Baf53a or Myc-Baf53b was confirmed by Western blot analysis at 2 days after Tet treatment. αTubulin was used as a loading control. The result is representative of three independent experiments.

Baf53b is a known homologue of Baf53a, and the protein structures of the two forms are very similar. They exhibit 91% similarity and 84% identity in amino acid sequences and 71% identity in nucleotide sequences (Supplemental Fig. 2C). In the present study, we examined whether Baf53b could rescue Baf53a-deficient ES cells from cell death. Figure 6A shows that overexpression of Baf53b, as well as Baf53a were able to rescue the viability observed in Baf53a-deficient ES cells. Crystal violet staining also revealed that colony forming capabilities were retained in Tet-treated, Baf53b-expressing, Baf53a cKO ES cells (Fig. 6B,C). Expression of Myc-tagged Baf53b in these samples were confirmed by Western blot analysis (Fig. 6D). Taken together, these results illustrate that Baf53b can rescue Baf53a-deficient ES cells from cell death.

### Baf53a-deficient ES cells overexpressing Baf53a or Baf53b exhibit maintenance of the undifferentiated state and increased proliferation

**Figure 7:**
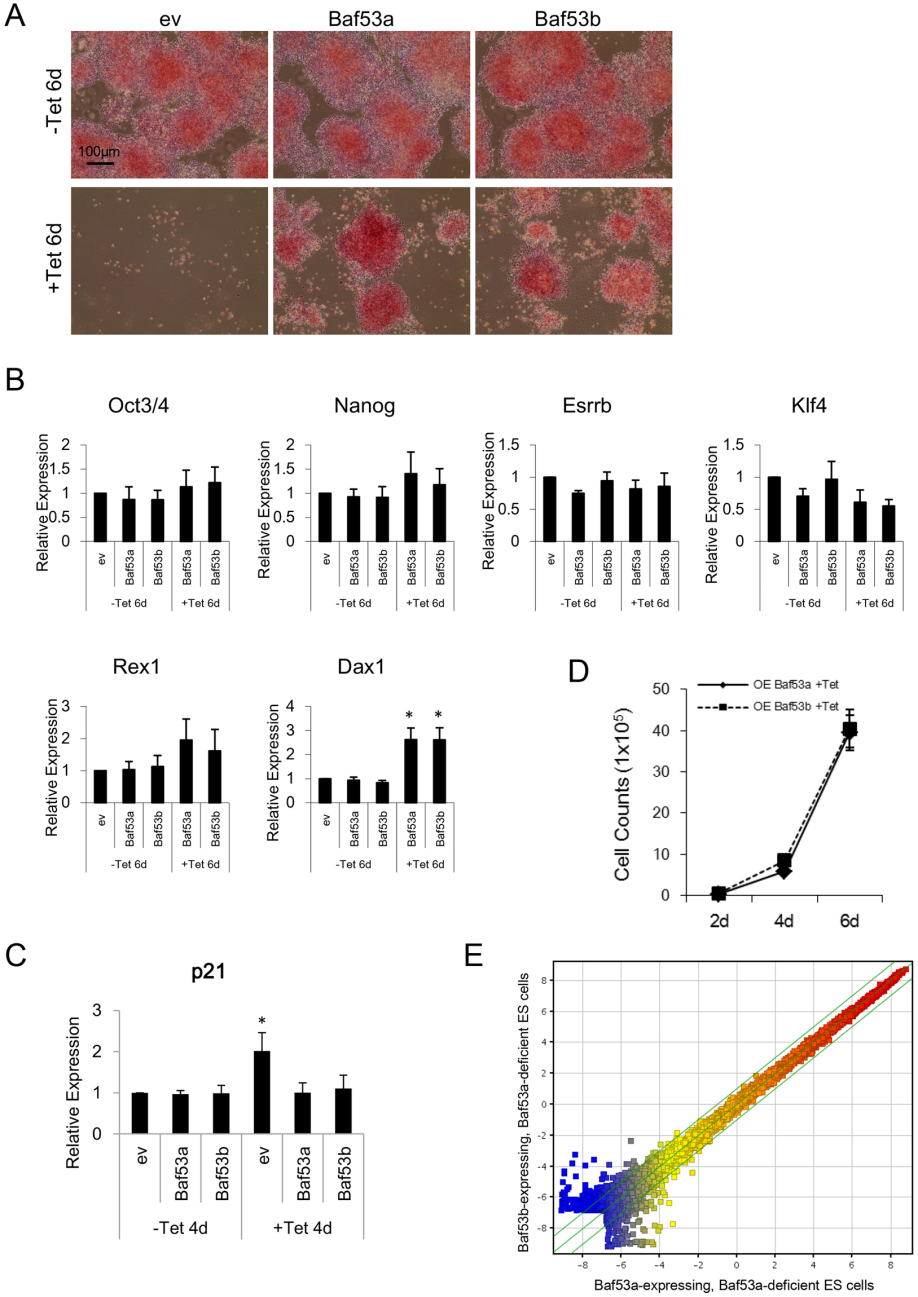
Baf53a- or Baf53b-expressing Baf53a-deficient ES cells exhibit the undifferentiated state. (**A**) Alkaline phosphatase staining of Baf53a- or Baf53b-expressing Baf53a-deficient ES cells. Baf53a cKO ES cells were transfected with expression vectors for empty (ev), Baf53a, or Baf53b. Transfected ES cells were divided into two dishes at 48 h after transfection and cultured with or without 1 μg/mL Tet. Alkaline phosphatase activity of these ES cells was detected by the VECTOR Red alkaline phosphatase substrate kit at 6 days after Tet treatment. Scale bar is 100 μm. (**B**) Expression of markers of undifferentiated cells in Baf53a- or Baf53b-expressing Baf53a cKO ES cells. Expression of Oct3/4, Nanog, Esrrb, Klf4, Rex1, and Dax1 mRNA in the indicated samples was examined by quantitative RT-PCR after 6 days. Because the Tet-treated, empty vector-transfected Baf53a cKO ES cells died after 6 days, mRNA expression was not examined. (**C**) Expression of p21 mRNA in Baf53a- or Baf53b-expressing Baf53a cKO ES cells. Expression of p21 mRNA in the indicated samples was examined by quantitative RT-PCR. The data in panels B and C were normalized to GAPDH expression. *p < 0.05 (t-test). (**D**) Growth curves of Baf53a- or Baf53b-transfected Baf53a-deficient ES cells. Baf53a- or Baf53b-transfected Baf53a-deficient ES cells were continually cultured in the presence of Tet and LIF and cell numbers were counted over time. The representative results shown are expressed as the means ± standard deviation of three independent experiments. Note: OE = overexpression. (**E**) DNA microarray analysis of Baf53a- and Baf53b-expressing, Baf53a-deficient ES cells. Scatter plots of log-ratios of relative expression levels were shown.

Finally, we examined the undifferentiated status of Baf53a- or Baf53b-expressing Baf53a-deficient ES cells. As shown in Fig. 7A, Baf53a- as well as Baf53b-expressing, Baf53a-deficient ES cells were AP positive. Quantitative RT-PCR analysis revealed that expression levels of undifferentiated state markers (Oct3/4, Nanog, Esrrb, Klf4, and Rex1) of Tet-treated Baf53a- or Baf53b-expressing Baf53a cKO ES cells were comparable to untreated cells and control cells. In particular, expression of Dax1 (another marker for the undifferentiated state) was enhanced in Tet-treated, Baf53a- or Baf53b-expressing, Baf53a cKO ES cells (Fig. 7B). These results indicate that overexpression of either Baf53a or Baf53b sustains the undifferentiated state in Baf53a-deficient ES cells.

In addition, we examined p21 mRNA expression in the rescued samples, as we previously observed p21 mRNA induction after Baf53a deletion (Fig. 4E). As shown in Fig. 7C, the increase in p21 mRNA expression in Tet-treated Baf53a cKO ES cells was reduced by overexpression of either Baf53a or Baf53b. Of note, we were able to continually culture Baf53a- or Baf53b-expressing, Baf53a-deficient ES cells in the presence of Tet, and cell counts revealed that proliferation was comparable between the two conditions (Fig. 7D). Finally, we performed a microarray analysis to compare global gene expression signatures between Baf53a- and Baf53b-expressing, Baf53a-deficient ES cells. As shown in Fig. 7E, global gene expression signatures were comparable between them. This result shows that Baf53a- or Baf53b-expressing, Baf53a-deficient ES cells exhibit similar gene expression profile. Together, these findings suggest that overexpression of either Baf53a or Baf53b rescues the reduced viability of Baf53a-deficient ES cells, and that Baf53b can compensate for Baf53a in ES cells.

## Discussion

In the present study, we discovered expression of Baf53a in ES cells even in differentiating conditions. Reduction of Baf53a mRNA expression was milder than other pluripotent marker genes. These results indicate that expression of Baf53a is indirectly regulated by LIF and/or Oct3/4. Mechanisms for direct regulation of Baf53a expression remain to be clarified. Importantly, we found that loss-of-expression of Baf53a leads to accumulation of p53 protein, induction of p21 mRNA, and activation of Caspase 3, resulting in reduced viability/survival of ES cells. Recently, Lu *et al*., demonstrated that knockdown of Baf53a expression by RNAi in ES cells decreased expression of pluripotent marker genes (Nanog and Oct3/4) and induced differentiation into primitive endoderm^34^, which suggested that functions of Baf53a included inhibition of differentiation in ES cells. Interestingly, our experiments showed that expression of Nanog and Oct3/4 mRNA, as well as of Oct3/4 protein, was comparable between control and Baf53a KD ES cells; Baf53a-deficient ES cells still expressed Nanog and Oct3/4 protein. Our two independent experiments (KO and KD) showed reduction of cell survival rather than loss of pluripotency. It is possible that phenotypes of gene knockdown differ because of differences for targets sequences, transfection condition, and so on. Therefore, we conclude that Baf53a is involved in survival, but not pluripotency, of ES cells, although esBAF complex facilitates pluripotency^27^. In addition, mRNA expression of Dax1, Nanog, and Esrrb were prone to induce after Baf53a deletion, suggesting that expression of them might be regulated by Baf53a directly or indirectly. Since protein expression levels of these undifferentiated specific markers were comparable between Baf53a KO and control ES cells, protein expression levels of these molecules might be stabilized by unknown post-translational regulation.

In support of the multifaceted role of Baf53a in ES cells, we found that p53, p21, and cleaved Caspase 3 were induced after Baf53a deletion. Since Baf53a-deficient ES cells express p53 protein and p21 mRNA, Baf53a is involved in survival of ES cells by repressing p53 activity. Regulation mechanisms of p53 by Baf53a remained to be clarified. Similar observations have also been reported in NIH3T3 cells, HeLa cells, and HSCs. For example, proliferation was repressed and p21 protein was induced in Baf53a KD NIH3T3 cells^37^, and p53 and p21 proteins were upregulated in Baf53a KD HeLa cells^38^. Cyclin-dependent kinase inhibitor, p21, is the downstream target of p53, and Baf53a has been shown to indirectly regulate p21 expression by interacting with p53 and repressing p53-mediated, p21-promoter activity^39^. Conditional deletion of the Baf53a gene in bone marrow cells revealed that Baf53a was required for proliferation of HSCs and survival of progenitor cells, and several p53 target genes were misregulated in Baf53a-deficient HSC compartments^32^. In particular, targeted disruption of the Baf53a gene led to early embryonic lethality; in intercrosses of heterozygous Baf53a mutant mice, no viable Baf53a-deficient fetuses were observed at E6.5 days^32^. ES cells are derived from inner cell mass/early epiblast from E3.5–E4.5 day-old embryos, thus Baf53a deficiency could explain the early embryonic lethality of the KO mice, highlighting the essential function of Baf53a in ES cells.

Pluripotency of ES cells is known to be enhanced by the esBAF complex^27^. Several pluripotency-associated transcription factors, including Oct3/4, Sox2, and Sall4, associate with the esBAF complex, especially with the component Brg1. Previously, we searched for Oct3/4-interacting proteins using yeast-two hybrid screening and identified Baf53a (unpublished data) and Dax1 as likely partners^10^. Additional comprehensive screening for Oct3/4-interacting proteins again identified Baf53a as a candidate factor^35^. Here, we showed an interaction between Baf53a and Oct3/4 using an MBP pulldown assay, and that Baf53a associated with the POU-specific domain of Oct3/4. Genome-wide chromatin immunoprecipitation analyses revealed that esBAF co-localized with Oct3/4 target genes in ES cells^21,40^; therefore, it is possible that Baf53a, as a critical component of the esBAF complex, recruits Oct3/4 and plays a role in enhancing pluripotency of ES cells. Notably, Baf53a was able to prevent differentiation of epidermal progenitors via downregulation of Klf4 and other epidermal differentiation genes^33^.

In addition to Oct3/4, Baf53a has been shown to interact with Tip60 (a component of the Tip60-p400 histone acetyltransferase complex) and c-Myc^41,42^. Tip60-p400 is required for the maintenance of ES cell-characteristics, and Tip60-deficient mice exhibited early embryonic lethality near the blastocyst stage, indicating essential function of Tip60 for cellular survival^43^. c-Myc-mediated oncogenic transformation is thought to be supported by Baf53a, and several deletion mutants of Baf53a inhibited the transformation^42^. Furthermore, c-Myc, Tip60, and Baf53a are members of a Myc-centered protein-protein interaction network in ES cells^44^. Importantly, Baf53a M3 mutant reduced interaction with either Tip60 or Myc, and attenuated incorporation into either the Tip60 HTA complex or the Myc-associated transcription complex, respectively^34,36^. In the present study, we demonstrated that Baf53a M3 mutant could not rescue the increased cell death observed in Baf53a-deficient ES cells. The impaired protein-protein interactions between Baf53a M3 and other molecules and the lack of cell survival of Baf53a-deficient ES cells with Baf53a M3, indicate that appropriate interactions among Baf53a, Tip60, and c-Myc are required for the survival of ES cells.

Interestingly, Baf53b, a homologue of Baf53a, rescued the phenotype of Baf53a-deficient ES cells; Baf53b-expressing, Baf53a-deficient ES cells proliferated and maintained their undifferentiated status, indicating redundant function between the two Baf53 forms. We found that Baf53b associated with the POU-specific domain of Oct3/4 (unpublished data). The 3 amino acid sequences that are mutated in Baf53a M3 is ERR (amino acids 388–390) in wild-type and ERK in Baf53b. Baf53b is expressed in post-mitotic neurons^45^ and is undetectable in ES cells (data not shown). Baf53b is assembled into the nBAF complex and is required for post-mitotic neuron development^25,46^. Notably, Baf53b-deficient mice exhibited perinatal lethality and exogenous expression of Baf53a failed to rescue the phenotype despite appropriate assembly of Baf53a into the nBAF complex^46^. Furthermore, a failure of dendritic development of Baf53b-deficient neuronal cells was rescued by Baf53b, but not by Baf53a. The subdomain 2 (amino acids 39–82) of Baf53a and Baf53b shows the lowest conservation between the two forms. An artificial chimeric protein, Baf53(b + a), where the subdomain 2 of Baf53a was replaced with that of Baf53b, rescued the dendritic development of Baf53b-deficient neurons^46^. In contrast, the effects of Baf53a deficiency on proliferation and undifferentiated status were restored by either Baf53a or Baf53b overexpression. Therefore, the extent of compensation between Baf53a and Baf53b appear to be cell and function dependent.

Overexpression of Baf53a in hepatocellular carcinoma was correlated with poor prognosis and metastasis^47^; expression of Baf53a in rhabdomyosarcoma was significantly higher than in normal muscle; suppression of Baf53a expression in rhabdomyosarcoma cells was associated with reduced proliferation^48^; and somatic mutation in the Baf53a gene was found in medulloblastomas^49^. In addition, Baf53a together with p63 were involved in oncogenesis of head and neck squamous cell carcinoma (HNSCC), and knockdown of Baf53a in HNSCC resulted in the suppression of sphere formation^50^. Recent studies indicated similarities between ES cells and cancer cells^51,52^ that could be mediated by a c-Myc regulatory network^44^. Furthermore, Baf53a is one of the molecules in a c-Myc-centered protein-protein interaction network^44^. These observations together with our findings suggest that Baf53a might be involved in regulating the characteristics observed in both ES cells and cancer cells.

Here, we demonstrated that Baf53a is involved in the survival of ES cells and that Baf53b can compensate for Baf53a in ES cells. Further investigation for each subunit of the esBAF complex will extend our understanding of the molecular mechanism that regulate proliferation, pluripotency, and the self-renewal capacities of ES cells.

## Material and Methods

### Cell culture

Mouse ES cell lines, E14tg2a (E14) ES cells, ZHBTc4 ES cells (Oct3/4 conditional knockout ES cells)^6^, and E14-derived Baf53a conditional knockout (cKO) ES cells, were cultured on gelatin-coated dishes in LIF-supplemented Dulbecco’s modified Eagle’s medium (DMEM) as described previously^4,53^. To regulate exogenous Oct3/4 or Baf53a expression, ZHBTc4 ES cells or Baf53a cKO ES cells, respectively, were cultured with or without 1 μg/mL Tet (Sigma-Aldrich, St. Louis, MO) for the indicated time. To induce differentiation, E14 ES cells were cultured without LIF for 3–6 days. Human embryonic kidney (HEK) 293 cells were cultured in DMEM containing 10% fetal bovine serum.

### Plasmid construction and transfection

To construct expression vectors, coding regions of either Baf53a or Baf53b were amplified by PCR using specific primers listed in Supplemental Table S1. Baf53a containing mutations at three amino acid residues (E388A/R389A/R390A), named Baf53a M3, was constructed by PCR using specific primers listed in Supplemental Table S1, using methods described before^36^. The coding regions of the genes were cloned into pCAGIP-Myc vector^54^, and these plasmids were referred to as pCAGIP-Myc-Baf53a, -Baf53b, and -Baf53a M3. pCMV5-Flag-MBP-Oct3/4 and its truncated mutants were constructed previously^10^.

For plasmid transfection of ES cells, Baf53a cKO ES cells were cultured in a 6 cm dish for 24 h, and then an expression vector (3 μg) were transfected into the cells by using Lipofectamine 2000 (Invitrogen, Carlsbad, CA) according to the manufacture’s protocol. After 48 h of transfection, these cells were divided into two new 6 cm dishes (0.5 × 10^5^ cells/dish) and were cultured with or without 1 μg/mL Tet for several days. Culture medium was replaced with fresh media once every 2 days. RNA and/or cell lysate samples were harvested from these cells and subjected to further examination.

### Knockdown of Baf53a expression

Double-stranded siRNAs were purchased from Eurofins Genomics (Tokyo, Japan). Their sequences were: siBaf53a #1: sense 5′-GGA CUG CCC UAA GGU UGA UUU-3′ and antisense 5′-AUC AAC CUU AGG GCA GUC CUC-3′; siBaf53a #2: sense 5′-GGC CAU UUU GGA UCA UAC AUA-3′ and antisense 5′-UGU AUG AUC CAA AAU GGC CUG-3′; and control EGFP: sense 5′-GCC ACA ACG UCU AUA UCA UGG-3′, and antisense 5′-AUG AUA UAG ACG UUG UGG CUG-3′. E14 ES cells were cultured in a 6 cm dish, and siRNA was transfected into ES cells using Lipofectamine 2000. Media was completely replaced after 24 h, and the cells were harvested after 48 h of transfection and subjected to further analysis.

### Generation of Baf53a conditional knockout ES cells

To generate Baf53a cKO ES cells, we used the CRISPR/Cas9 system. The targeting site (5′-GTT GAT TTC CCC ACG GCT AT-3′) of the Baf53a gene was selected using the CRISPR program <http://crispr.dbcls.jp/>. pX330-U6-Chimeric_BB-CBh-hSpCas9 was a gift from Feng Zhang (Addgene plasmid # 42230)^55,56^. Oligonucleotides for the target sequence were cloned into the BbsI sites of the plasmid (pX330- Baf53a).

For construction of the Tet-inducible Baf53a expression vector, we first generated a CRISPR/Cas9-resistant Baf53a mutant (Baf53a^R^) cDNA by PCR using the primers listed in Table S1. For Baf53a^R^, the targeting site of the Baf53a cDNA was changed into 5′-GTT GAc TTt CCt ACc GCc AT-3′, which still kept the amino acid sequences intact. The coding region of Baf53a^R^ cDNA was then cloned into a Tet-inducible/puromycin-resistant pTRE-tTA2p vector, which was modified from pTRE-tTA2p-myc^8^; the resulting plasmid was named pTRE-tTA2p-Baf53a^R^. pTRE-tTA2p-Baf53a^R^ was transfected into E14 ES cells and these cells were cultured in the presence of puromycin for 10 days. After establishment of a Baf53a^R^ inducible ES cell clone (E14-Tet-Baf53a^R^ #23), pX330-Baf53a was transfected into the clone #23 in the absence of Tet (*i.e*., exogenous Baf53a^R^ expressing condition). Seven homozygous-mutant clones were obtained, and clone #46, which had a 23 bp deletion in one allele and a 7 bp deletion in the other allele, was further analyzed as our Baf53a cKO ES cells.

### Quantitative RT-PCR, Western blot analysis and MBP pull-down assay

Quantitative RT-PCR was performed as described previously^13^. Briefly, cDNAs was synthesized from total RNA of ES cells by using ReverTra Ace (Toyobo, Osaka, Japan) and oligo (dT)_12–18_ primers (Nippon EGT, Toyama, Japan). Quantitative RT-PCR using SYBR green was performed with an Agilent Technologies Stratagene Mx3005 P Real-Time PCR System (Stratagene, La Jolla, CA). The expression levels of target genes were normalized with glyceraldehyde-3-phosphate dehydrogenase (GAPDH). Primers sequences were described before^7,11,12^ or listed in Supplemental Table S1.

For Western blot analysis, cells were lysed in a cell lysis solution (20 mM HEPES, 10 mM MgCl_2_, 1 mM EDTA, 10 mM sodium fluoride, 25 mM β-glycerophosphate, 1 mM sodium orthovanadate, 20 µg/mL aprotinin, 10 µg/mL leupeptin, 10 µg/mL pepstatin A, 1% TritonX-100, and 10% Glycerol), and these samples were subjected to Western blot analysis using anti-Baf53a (A301-391A, Bethyl Laboratories), anti-Nanog (Repro CELL, Yokohama, Japan), anti-Oct3/4 (sc-9081; Santa Cruz Biotechnology), anti-Dax1 (39984; Active Motif, CA), anti-Esrrb (PP-H6705-00; Perseus Proteomics, Tokyo, Japan), anti-p53 (Leica Biosystems, Germany), anti-Caspase 3 (9662 S, Cell Signaling Technology), anti-cMyc (sc-40, Santa Cruz Biotechnology), anti-PCNA (MH-12-3, MBL, Japan), and anti-αTubulin (MBL, Japan) primary antibodies followed by horseradish peroxidase-conjugated anti-mouse or anti-rabbit secondary antibodies (Millipore, Billerica, MA). The blot was visualized using enhanced chemiluminescence reagents (PerkinElmer, Waltham, MA) and an LAS-1000 image analyzer (Fuji Film, Tokyo, Japan).

An MBP pulldown assay was performed as described previously^10^. Briefly, pCAGIP-Myc-Baf53a was co-transfected with either pCMV5-Flag-MBP-Oct3/4 or its truncated mutants, including C-terminal region, N-terminal region, POU domain, POU-homeo domain, and POU-specific domain, into HEK293 cells. Two days after transfection, cell lysates were harvested and incubated overnight at 4 °C with amylose resin. The beads were washed three times with a washing buffer (50 mM Tris-HCl [pH 7.5], 2 mM MgCl_2_, 150 mM NaCl), and the bound proteins were eluted by boiling in sodium dodecyl sulfate (SDS) sample buffer and subjected to Western blot analysis using anti-cMyc or anti-Flag (F3165; Sigma-Aldrich, St. Louis, MO) antibodies. Original full blot images are shown in Supplemental Fig. 3.

### Crystal violet staining and AP staining

ES cells were washed with PBS (−) and the cells were stained by 0.1% crystal violet-50% methanol solution. Cell colonies were imaged by a scanner, and then lysed in a solution containing 1.0% SDS. An absorbance of the lysate was measured with a wavelength of 600 nm.

For AP staining, ES cells were washed with PBS (−), fixed with 4% formalin at room temperature for 20 min. After washing the cells with PBS (−), AP activity was determined using a vector blue/red AP substrate kit (Vector Laboratories, Inc., Burlingame, CA) according to the manufacture’s protocol.

### Cell proliferation assay

The number of viable cells was measured by a direct cell count and the WST-1 assay. For the WST-1 assay, ES cells were transferred into a 96-well plate and after 2 days of culture, 10 μL of WST-1 reagent (Roche Diagnostics, Germany) was added to the culture medium. The cells were then incubated for 30 min in a humidified atmosphere (37 °C, 5% CO_2_). The plate was thoroughly shaken for 1 min on a microplate reader (Tecan, Mannedorf, Switzerland) and the absorbance of the culture medium was measured at wavelengths of 450 nm and 630 nm.

### Immunofluorescent stain

Baf53a cKO ES cells were cultured with or without Tet for 4 days in 24-well plate. Cells were washed by 1 x PBS (−) and fixed by 10% formalin solution for 30 min at room temperature. These cells were washed with 0.2% Triton X-100 in PBS (−) for 3 times and then incubated with 5% FBS-0.2% Triton X-100 in PBS (−) at 37 °C for 1 hour. The cells were then incubated with 50-fold diluted anti-Ki67 antibody (ab15580, Abcam, Cambridge, UK) at 4 °C for overnight, followed by incubation with 100-fold diluted goat anti-rabbit IgG FITC (Santa Cruz Biotechnology). Vectashield mounting medium (Vector Laboratories, Inc. Burlingame, CA) was used for DAPI staining.

### DNA Microarray analysis

Baf53a cKO ES cells were transfected with expression vectors for Baf53a or Baf53b. Forty-eight hours after transfection, these cells were cultured with 1 μg/mL Tet for 6 days. One pair RNA was isolated from these ES cells. Cyanine-3 (Cy3) labeled cRNA was prepared from 0.2 μg RNA using the Low Input Quick Amp Labeling Kit (Agilent Technologies) according to the manufacturer’s instructions, followed by RNeasy column purification (QIAGEN, Valencia, CA). Samples were hybridized to Whole Mouse Genome microarray 4 × 44 K Ver. 2.0 (G2519F, Agilent Technologies). The slides were scanned on the Agilent DNA Microarray Scanner (G2539A) using one color scan setting for 4 × 44k array slides. The scanned images were analyzed with Feature Extraction Software 11.0.1.1 (Agilent) using default parameters to obtain background subtracted and spatially detrended Processed Signal intensities. The raw data has been deposited in the Gene Expression Omni-bus (GEO) database, under number GSE103694.

### Statistical analysis

Statistical analyses were performed using two-tailed Student’s t-tests. Values of p < 0.05 were considered significant. Data were indicated as means ± standard deviation.

## Electronic supplementary material

Supplemental data
