# Supplementary material for: Baf53a is involved in survival of mouse ES cells, which can be compensated by Baf53b
Source: Sci Rep. 2017 Oct 25;7:14059. doi: 10.1038/s41598-017-14362-4 (PMC5656580; doi:10.1038/s41598-017-14362-4)
Supplement: Supplementary file 1 — Supplemental data [file 41598_2017_14362_MOESM1_ESM.pdf]

Supplemental Information

Baf53a is involved in survival of mouse ES cells, which can be compensated by Baf53b

Bo Zhu*[1], Atsushi Ueda*[1], Xiaohong Song[1], Shin-ichi Horike[2], Takashi Yokota[#1] & Tadayuki Akagi[#1]

[1]Department of Stem Cell Biology, Faculty of Medicine, Institute of Medical, Pharmaceutical and Health Sciences, and [2]Advanced Science Research Center, Kanazawa University. 13-1 Takara-machi, Kanazawa, Ishikawa 920-8640, Japan

*B.Z. and A.U. contributed equally in this manuscript.

#Correspondence and request for materials should be addressed to T.Y. (tyokota@med.kanazawa-u.ac.jp) and T.A. (tadayuki@staff.kanazawa-u.ac.jp)

Supplemental Table S1. List of primer sequences for RT-PCR

Gene Name		Sequences
For RT-PCR		
Baf53a	F	TAGTTTCCAGGCCATTTTGG
	R	GTGGTGTGGGTAGCTCCACT
Nanog	F	AGGGTCTGCTACTGAGATGCTCTG
	R	CAACCACTGGTTTTTCTGCCACCG
p21	F	GAACGGTGGAACTTTGACTTC
	R	AGAGTGCAAGACAGCGACAAG
For cloning		
Baf53a full	F	CAATTGATGAGCGGCGGCGTGTACGGC
	R	GCGGCCGCTCAAGGGCATTTTCTTTCTACA
Baf53b full	F	GAATTCATGAGCGGGGGCGTCTAC
	R	GCGGCCGCTCAGGGGCACTTCCGCTC
Baf53a M3	F	GACGGTGGCGGCAGCTTTCAGCT
	R	AGCTGAAAGCTGCCGCCACCGTC
Baf53a^{R}	F	GGTTGACTTTCCTACCGCCATCGGTGTGGTGCTGGAGAGA
	R	ACCGATGGCGGTAGGAAAGTCAACCTTAGGGCAGTCCTCG

Legends for Supplemental Figures

Supplemental Figure 1. Baf53a knockdown in ES cells induces the expression of undifferentiated state marker genes and reduces ES cell proliferation. (A) Alkaline phosphatase stain in control and Baf53a knockdown ES cells. EGFP control siRNA (Control) or two independent Baf53a siRNAs (siBaf53a #1 and #2) were transfected into E14 ES cells. Alkaline phosphatase activity of these ES cells was detected by VECTOR Blue alkaline phosphatase substrate kit 48h after transfection and morphological features were observed. Scale bar is 250 μm. (B) Expression of undifferentiated state marker genes in Baf53a knockdown ES cells. Expression of Baf53a, Dax1, Oct3/4, Esrrb, and Nanog mRNA in control, siBaf53a #1, or siBaf53a #2 ES cells was examined by quantitative RT-PCR. (C) Expression of Oct3/4 protein in Baf53a knockdown ES cells. Protein expression of Baf53a and Oct3/4 in control, siBaf53a #1, or siBaf53a #2 ES cells was examined by Western blot. αTubulin was used as a loading control. The result is representative of three independent experiments. (D) Proliferation of Baf53a knockdown ES cells. Control, siBaf53a #1, or siBaf53a #2 ES cells were seeded at 500, 1000, or 3000 cells per well in a 96-well dish, and subjected to WST-1 assay at 2 days after incubation. The result is representative of three independent experiments represents the means ± standard deviations of triplicate assays.

Supplemental Figure 2. (A) E14 ES cells were transfected with expression vectors for empty (ev), Baf53a WT, or Baf53a M3. Transfected ES cells were cultured in the presence of 1μg/mL puromycin; and these cells were subjected to the WST-1 assay. The data is representative of three independent experiments. (B) Expression of exogenous Myc-

tagged Baf53a protein. Expression of either Myc-Baf53a WT or Myc-Baf53a M3 in the panel A was confirmed by Western blot analysis. αTubulin was used as a loading control. The result is representative of three independent experiments. (C) Amino acid sequence alignment of Baf53a and Baf53b. Baf53a and Baf53b consist of 429 and 426 amino acid residues, respectively, and they exhibit 84% identity of these two amino acid sequences <http://web.expasy.org/sim/>. The three amino acids (ERR) of Baf53a to construct Baf53a M3 are shown as red letters.

Supplemental Figure 3. Original full blot images of Western blot analyses. (A) Original images of Figure 1C. (B) Original images of Figure 3A. (C) Original images of Figure 4D. (D) Original images of Figure 4F. (E) Original images of Figure 5E. (F) Original images of Figure 6D. (G) Original images of Supplemental Figure 1C. (H) Original images of Supplemental Figure 2B.

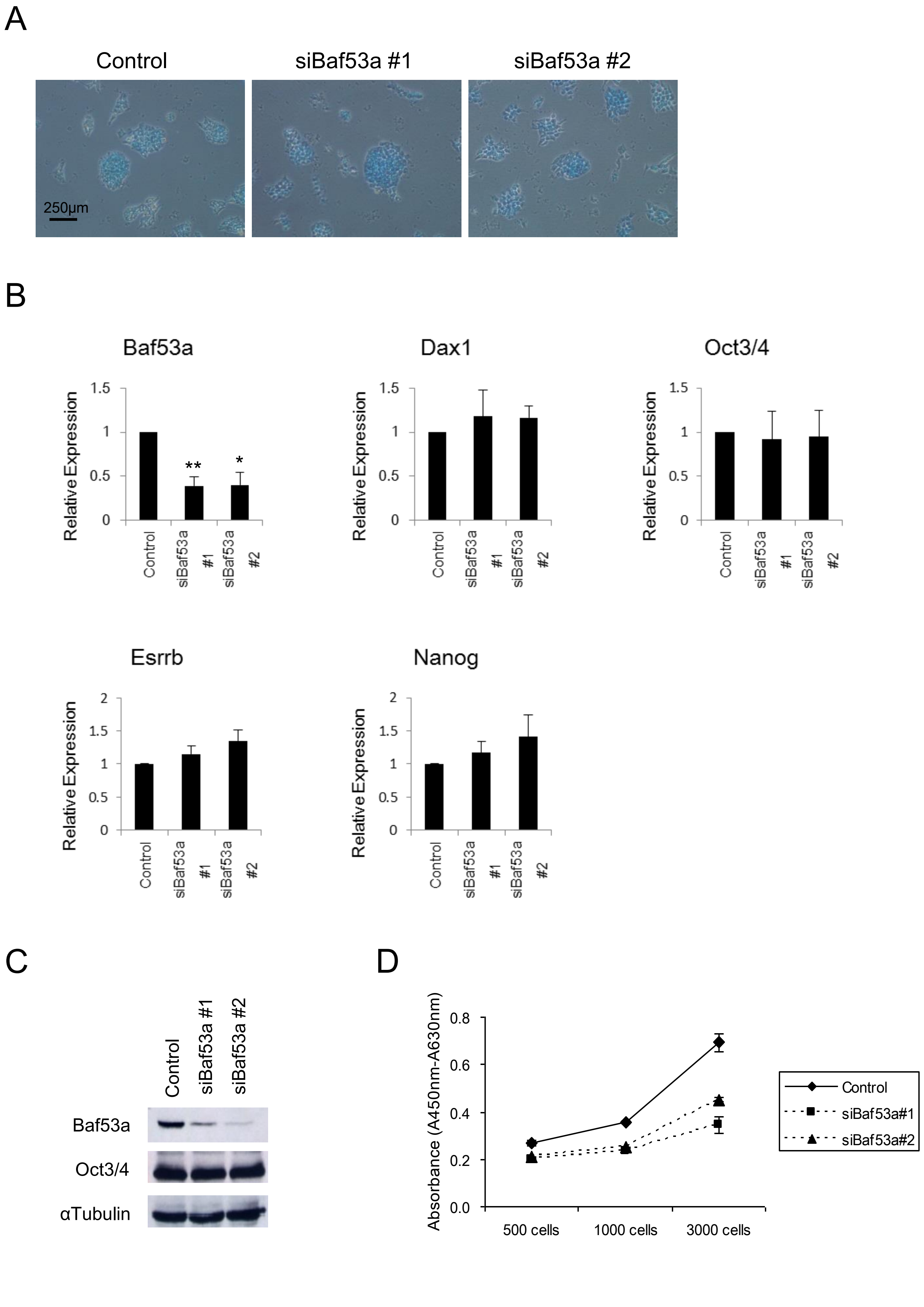

Supplemental Figure 1

A

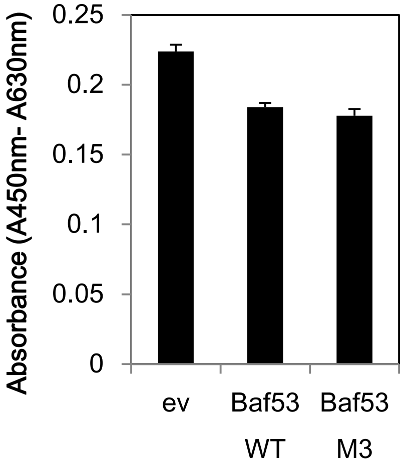

B

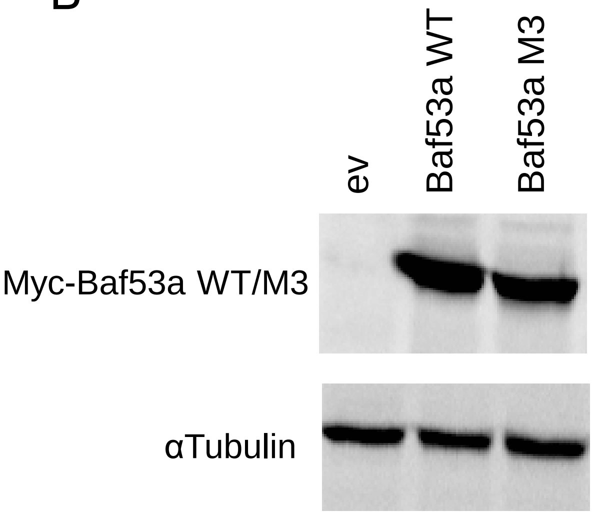

C

```
Baf53a     1 MSGGVYGGDEVGALVFDIGSYTVRAGYAGEDCPKVDFPTAIGVVLERDDGSTMMEIDGDK
Baf53b     1 MSGGVYGGDEVGALVFDIGSFSVRAGYAGEDCPKADFPTTVGLLAAEEGGG--LELEGEK
             ********************  ************ ****  *       *    *  * *

Baf53a    61 GKQGGPTYYIDTNALRVPRENMEAISPLKNGMVEDWDSFQAILDHTYKMHVKSEASLHPV
Baf53b    59 EKKG-KIFHIDTNALHVPRDGAEVMSPLKNGMIEDWECFRAILDHTYSKHVKSEPNLHPV
              * *     ****** ***   *  ******* ***  * *******  *****  ****

Baf53a   121 LMSEAPWNTRAKREKLTELMFEHYSIPAFFLCKTAVLTAFANGRSTGLILDSGATHTTAI
Baf53b   118 LMSEAPWNTRAKREKLTELMFEQYNIPAFFLCKTAVLTAFANGRSTGLVLDSGATHTTAI
             ********************** * *********************** ***********

Baf53a   181 PVHDGYVLQQGIVKSPLAGDFITMQCRELFQEMNIELIPPYMIASKEAVREGSPANWKRK
Baf53b   178 PVHDGYVLQQGIVKSPLAGDFISMQCRELFQEMAIDIIPPYMIAAKEPVREGAPPNWKKK
             ********************** ********** *  ******* ** **** * *** *

Baf53a   241 EKLPQVTRSWHNYMCNCVIQDFQASVLQVSDSTYDEQVAAQMPTVHYEFPNGYNCDFGAE
Baf53b   238 EKLPQVSKSWHNYMCNEVIQDFQASVLQVSDSPYDEQVAAQMPTVHYEMPNGYNTDYGAE
             ******  ******** *************** *************** ***** * ***

Baf53a   301 RLKIPEGLFDPSNVKGLSGNTMLGVSHVVTTSVGMCDIDIRPGLYGSVIVAGGNTLIQSF
Baf53b   298 RLRIPEGLFDPSNVKGLSGNTMLGVGHVVTTSIGMCDIDIRPGLYGSVIVTGGNTLLQGF
             ** ********************** ****** ***************** ***** * *

Baf53a   361 TDRLNRELSQKTPPSMRLKLIANNTTVERRFSSWIGGSILASLGTFQQMWISKQEYEEGG
Baf53b   358 TDRLNRELSQKTPPSMRLKLIASNSTMERKFSPWIGGSILASLGTFQQMWISKQEYEEGG
             ********************** * * ** ** ***************************

Baf53a   421 KQCVERKCP
Baf53b   418 KQCVERKCP
             *********
```

Supplemental Figure 2

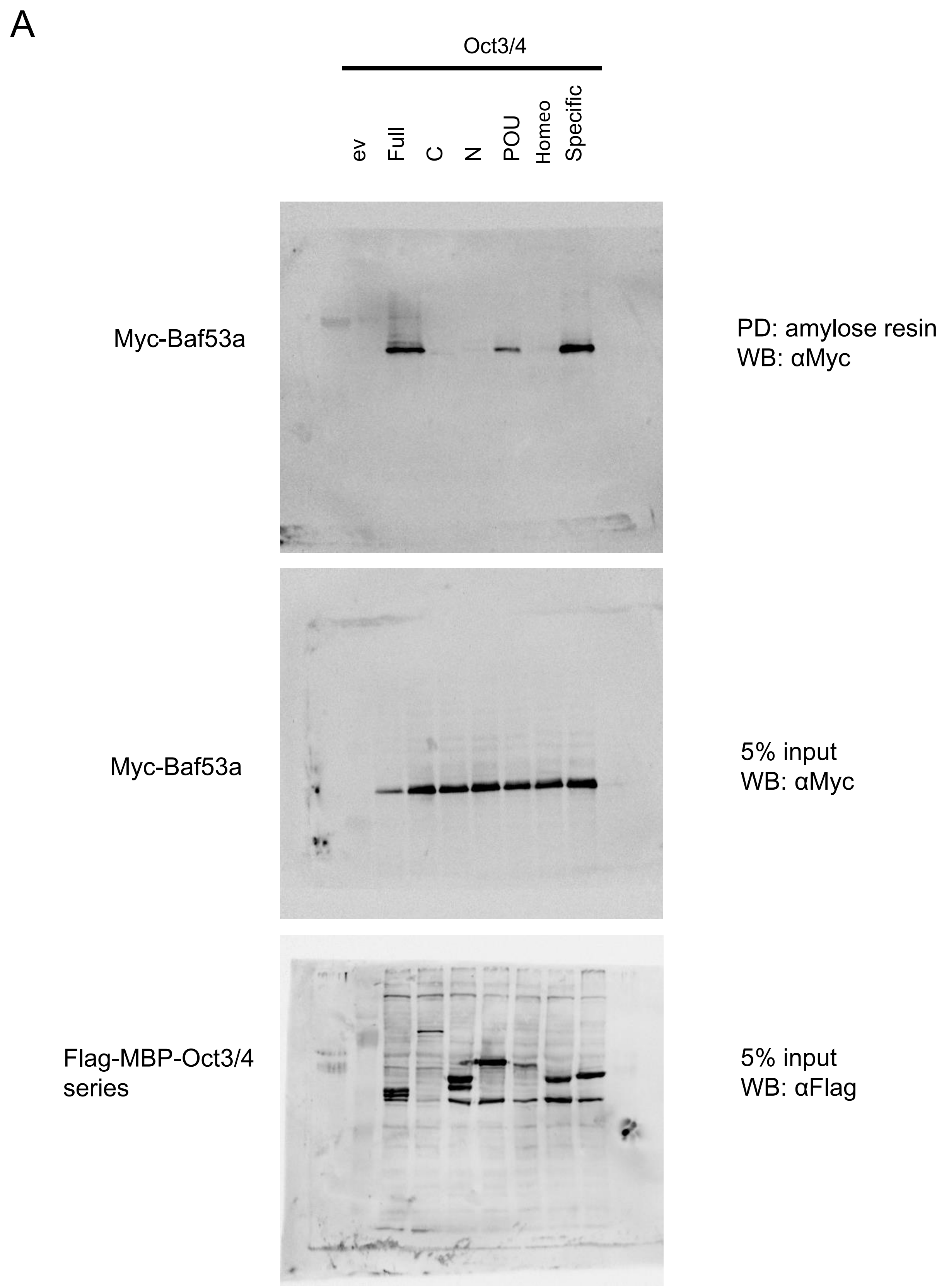

Supplemental Figure 3A

B

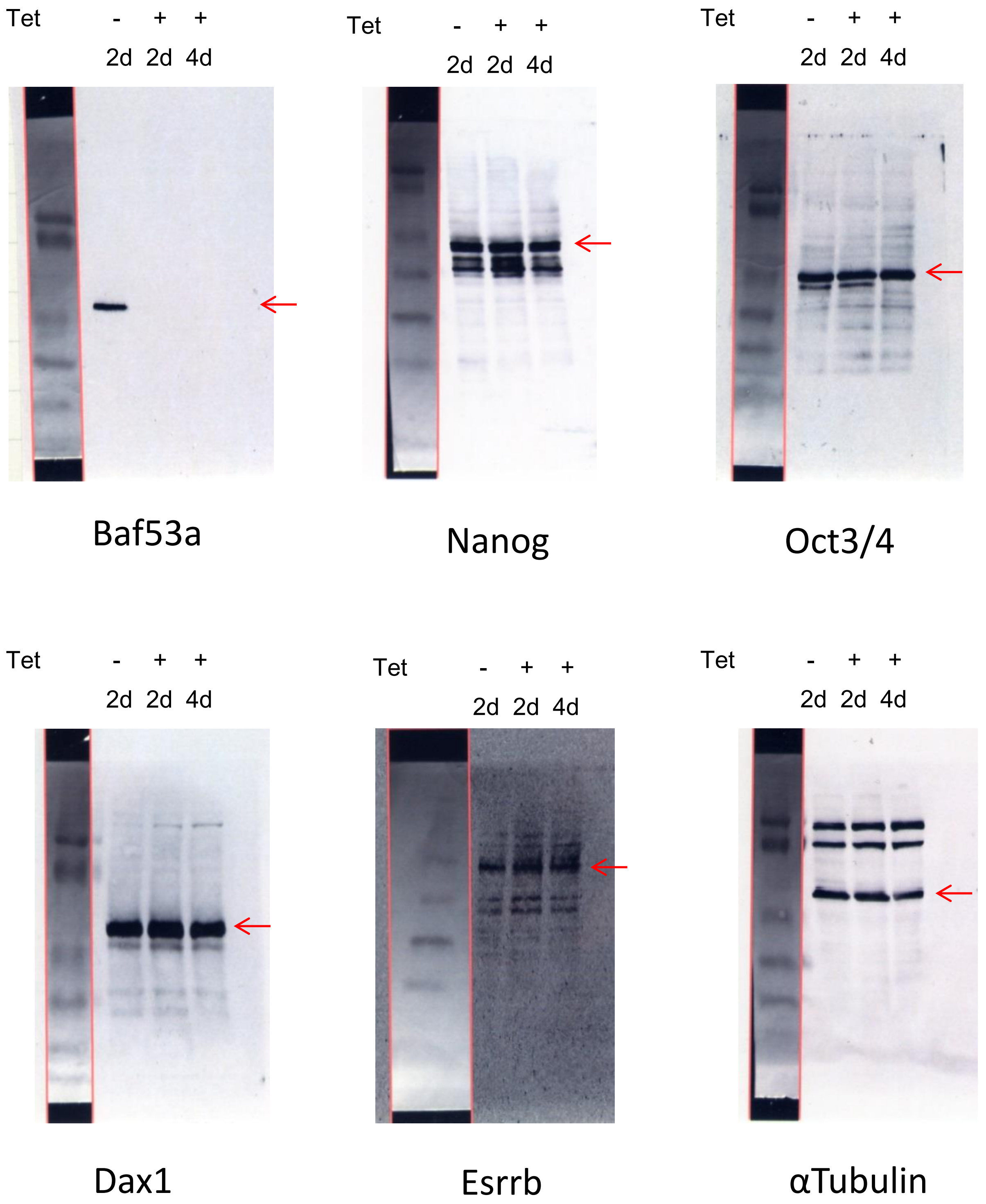

Supplemental Figure 3B

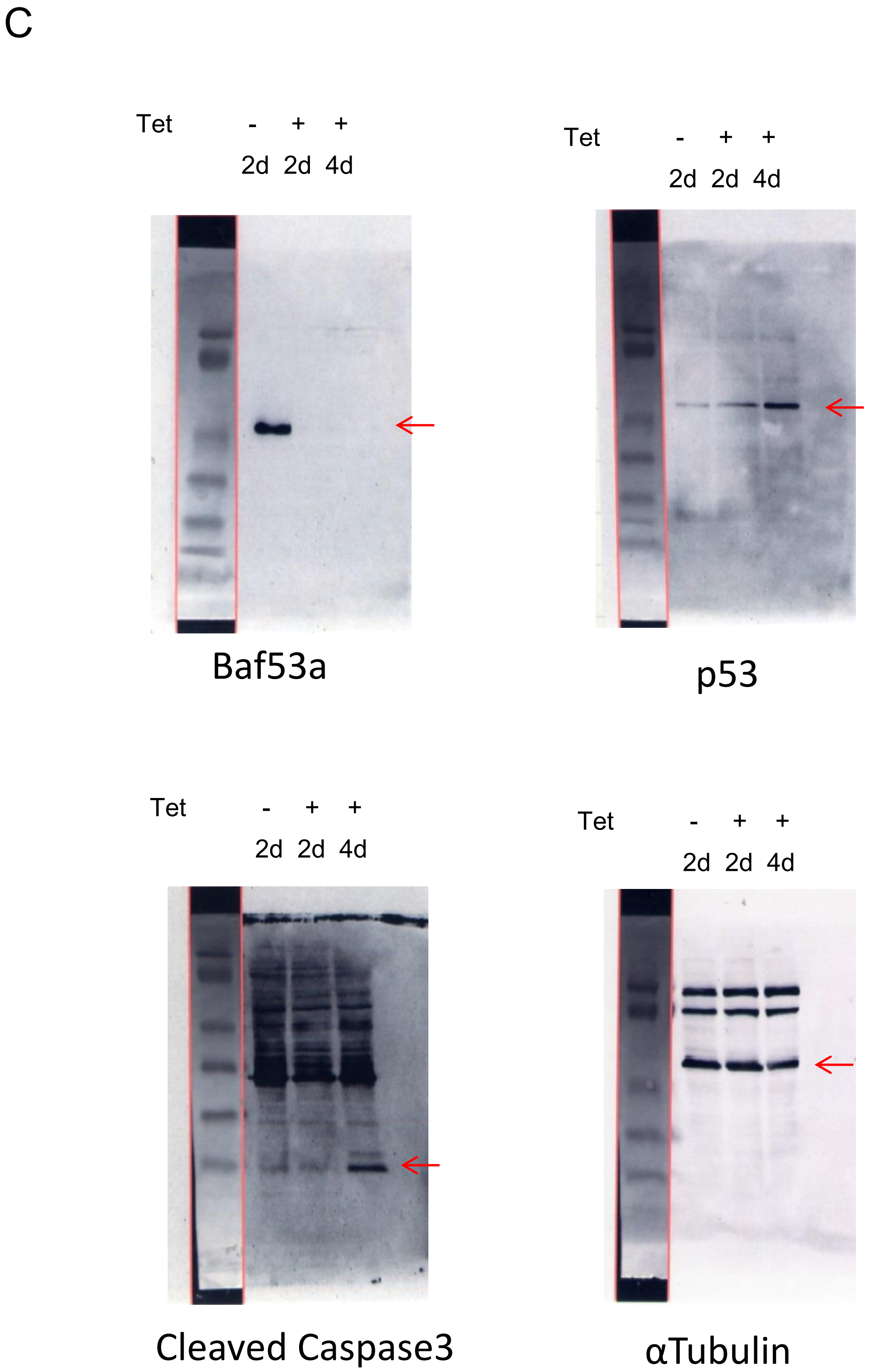

Supplemental Figure 3C

D

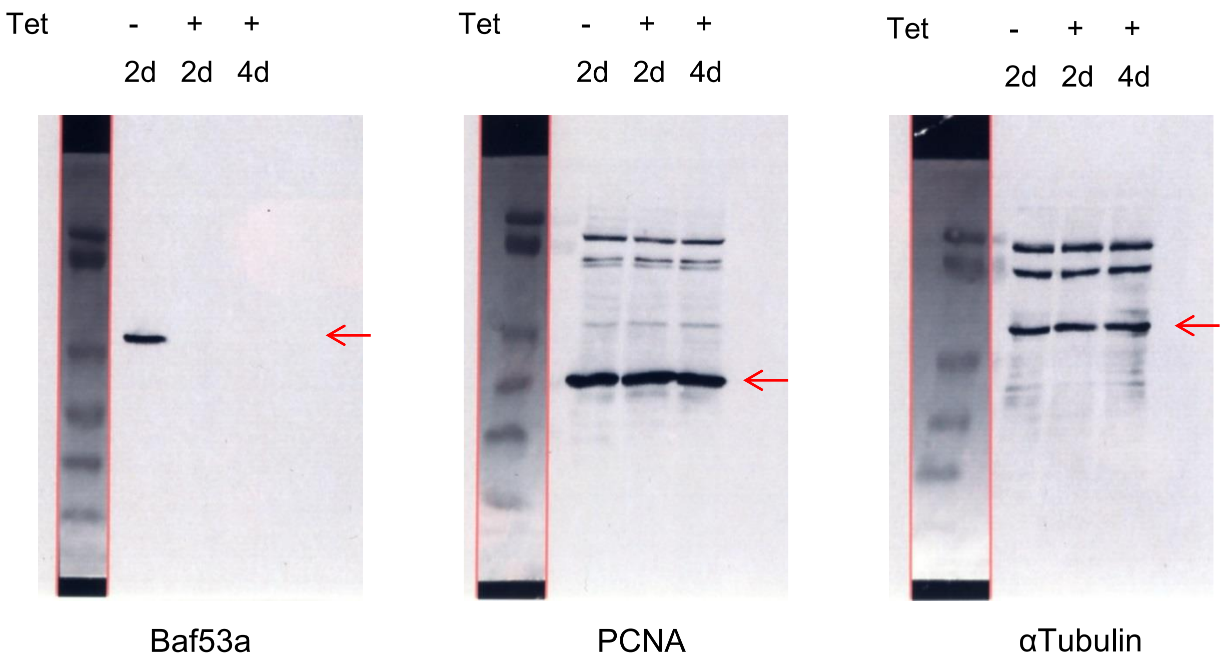

E

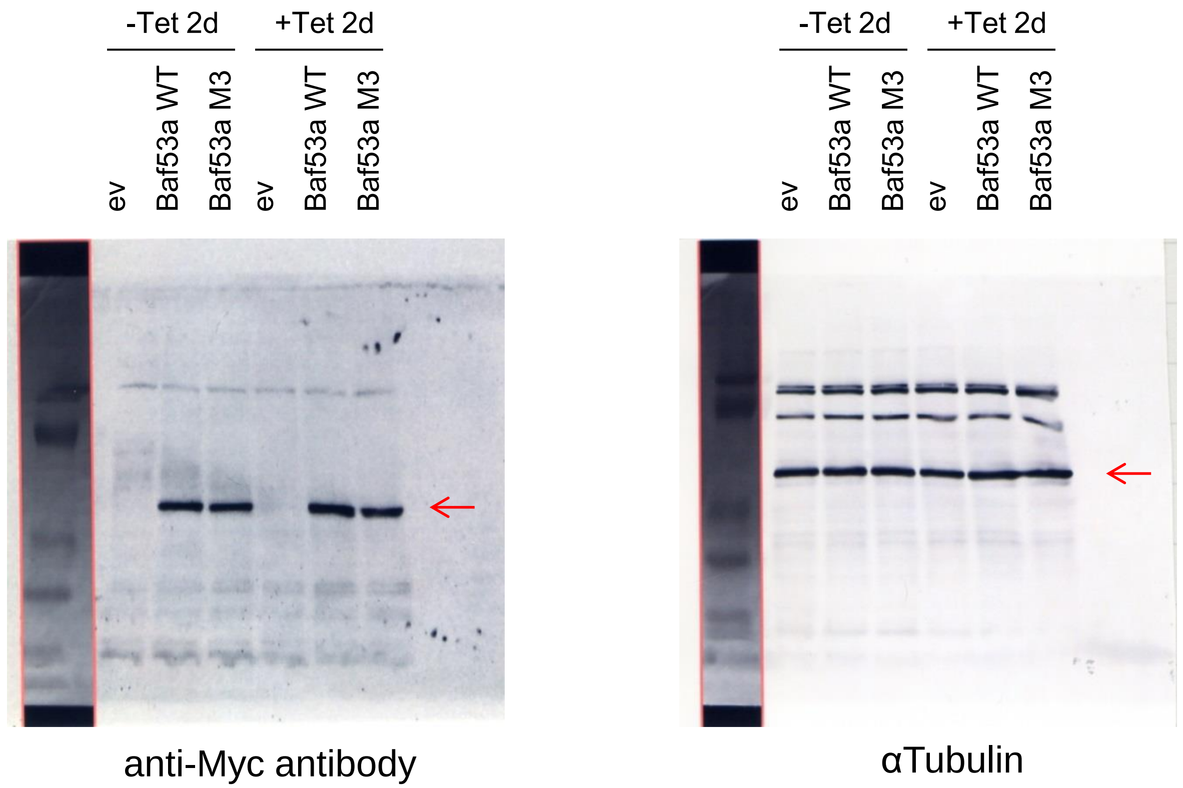

Supplemental Figure 3DE

F

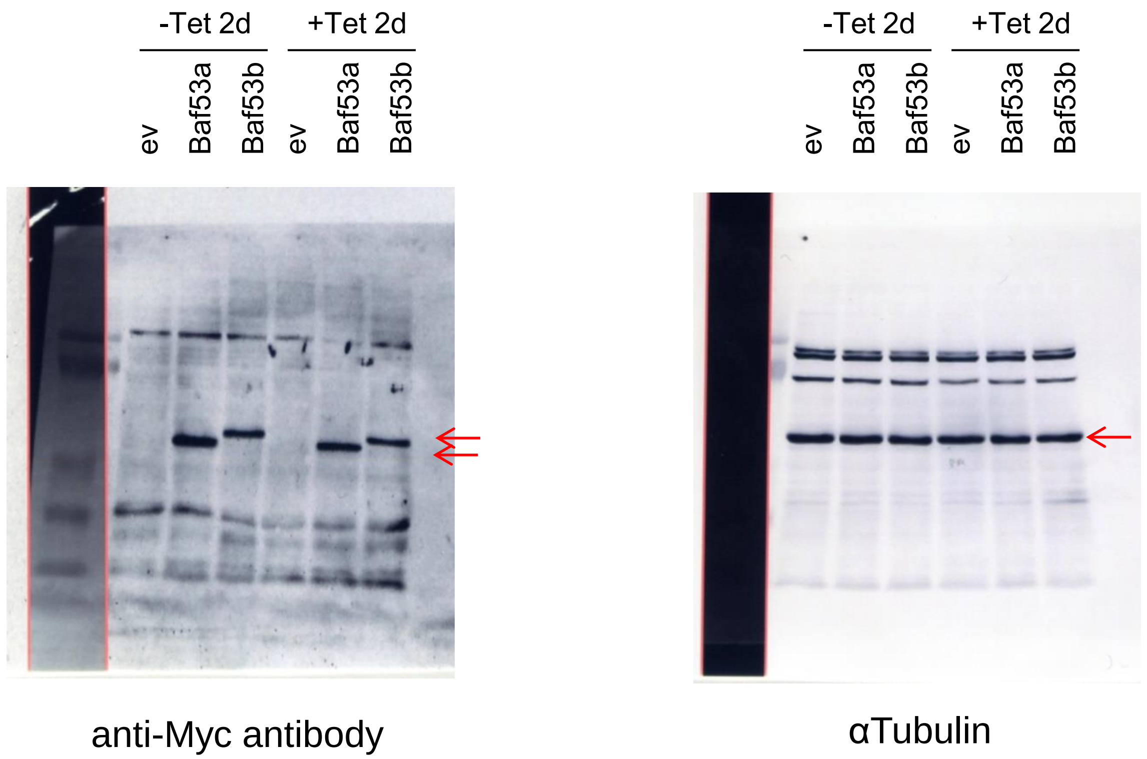

G

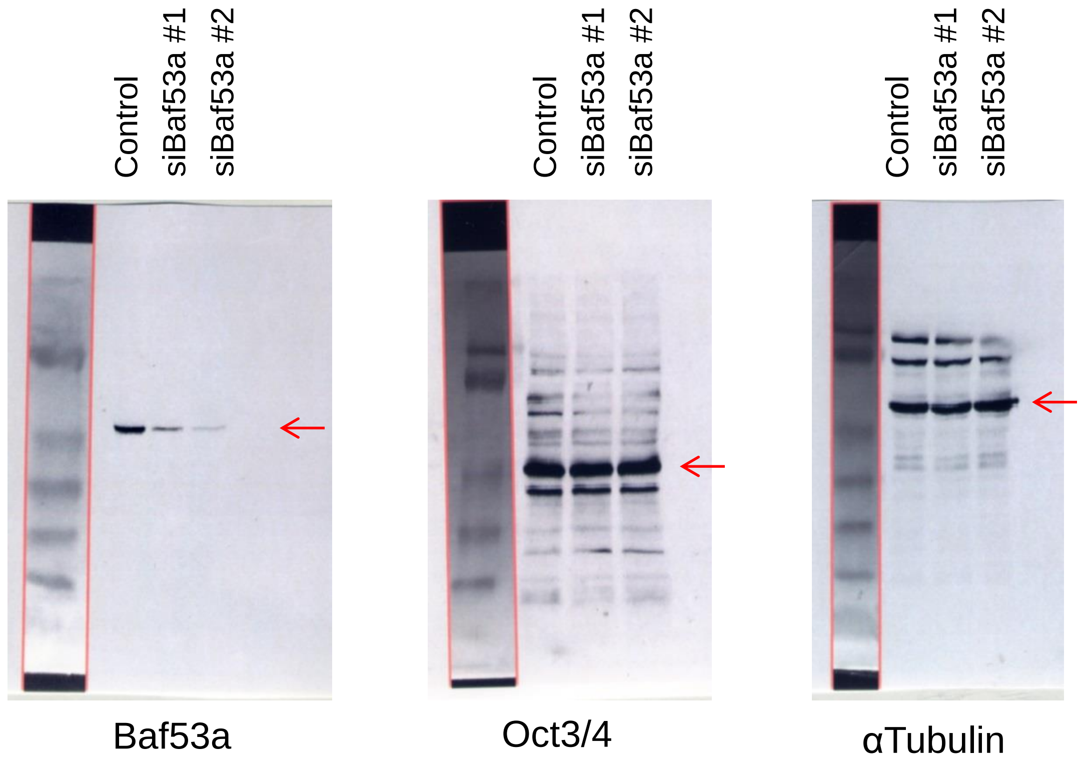

Supplemental Figure 3FG

H

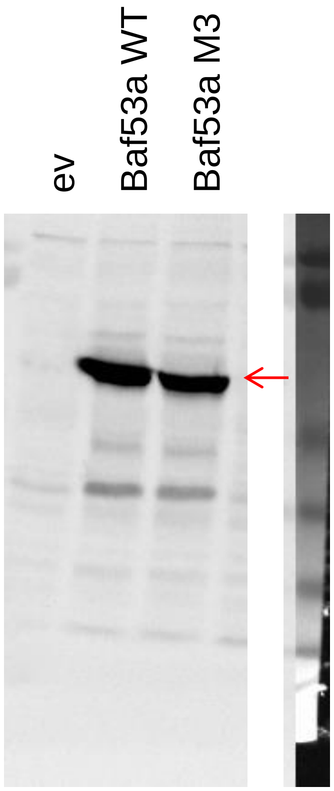

anti-Myc antibody

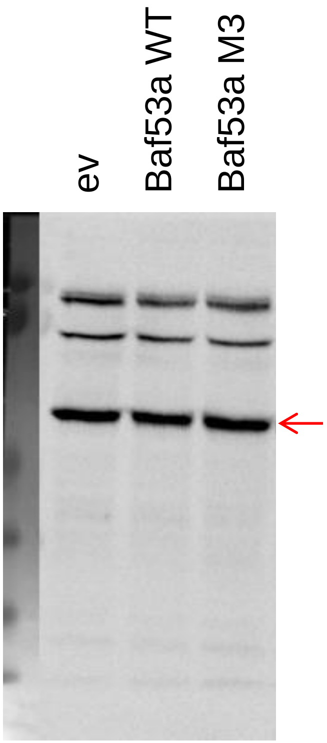

αTubulin

Supplemental Figure 3H
